# AutomataGPT: Transformer‐Based Forecasting and Ruleset Inference for Two‐Dimensional Cellular Automata

**DOI:** 10.1002/advs.202511352

**Published:** 2026-04-09

**Authors:** Jaime A. Berkovich, Noah S. David, Markus J. Buehler

**Affiliations:** ^1^ Laboratory for Atomistic and Molecular Mechanics (LAMM) Department of Materials Science and Engineering Massachusetts Institute of Technology Cambridge Massachusetts USA; ^2^ Laboratory for Atomistic and Molecular Mechanics (LAMM) Massachusetts Institute of Technology Cambridge Massachusetts USA; ^3^ Laboratory for Atomistic and Molecular Mechanics (LAMM) Department of Civil and Environmental Engineering Massachusetts Institute of Technology Cambridge Massachusetts USA; ^4^ Department of Mechanical Engineering Schwarzman College of Computing Massachusetts Institute of Technology Cambridge Massachusetts USA; ^5^ Center for Computational Science and Engineering, Schwarzman College of Computing Massachusetts Institute of Technology Cambridge Massachusetts USA

**Keywords:** cellular automata, forecasting, generative pretrained transformers, interpretable modeling, rule inference

## Abstract

Cellular automata (CA) provide a minimal formalism for investigating how simple local interactions generate rich spatiotemporal behavior in domains as diverse as traffic flow, ecology, tissue morphogenesis, and crystal growth. However, automatically discovering the local update rules for a given phenomenon and using them for quantitative prediction remains challenging. Here we present AutomataGPT, a decoder‐only transformer pretrained on ∼1 million simulated trajectories that span 100 distinct two‐dimensional binary deterministic CA rules on toroidal grids. When evaluated on previously unseen rules drawn from the same CA family, AutomataGPT attains 98.5% perfect one‐step forecasts and reconstructs the governing update rule with up to 96% functional (application) accuracy and 82% exact rules‐matrix match. These results demonstrate that large‐scale pretraining over wider regions of rule space yields substantial generalization in both the forward (state forecasting) and inverse (rule inference) problems, without hand‐crafted priors. By showing that transformer models can faithfully infer and execute CA dynamics from data alone, our work lays the groundwork for constructing interpretable CA surrogates for systems whose coarse‐grained dynamics are reasonably approximable within a chosen local CA rule class, opening avenues in biology, tissue engineering, physics, and AI‐driven scientific discovery.

## Graphical abstract

We introduce AutomataGPT, a generative pretrained transformer (GPT) trained on synthetic spatiotemporal data from 2D cellular automata to learn symbolic rules. Demonstrating strong performance on both forward and inverse tasks, AutomataGPT establishes a scalable, domain‐agnostic framework for interpretable modeling, paving the way for future applications to real physical systems across science and engineering.

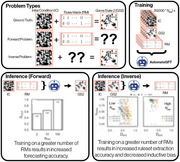

## 1 Main

**FIGURE 1:**
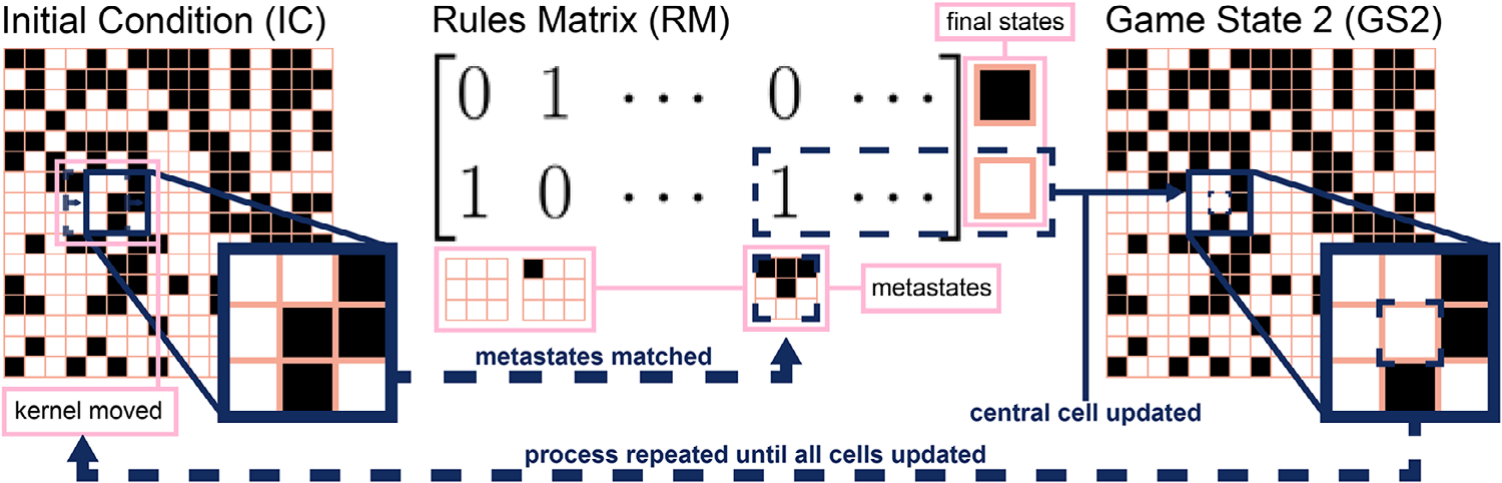
Illustration of the process of computing one future global state (or “game state”) for a 2D binary deterministic r=1 cellular automaton, based on an inputted initial 16×16 binary grid representing an initial global state/initial condition (IC) and 2D binary array representing local state transition rules, referred to here as a “rules matrix” (RM). Local states are updated based on a 3×3 kernel (moving window) that scans the IC and matches observed cell‐neighborhood pairs (metastates) with their corresponding columns in the RM. For a given metastate's RM column, a row is selected if it contains a 1, indicating 100% probability of state transition. The selected row corresponds to the state to which the cell in the center of the kernel is reassigned. Note: for this study, we consider toroidal grids (2D grids with periodic boundary conditions).

Cellular automata (CA) are a class of algorithms that consist of an array of cells, each having a single “state” out of a finite set of possible states, with recursive, local update rules governing state transitions at discrete time intervals (Figure 1). These algorithms have been of particular interest for computer scientists and natural scientists alike, due to their ability to exhibit complex, emergent phenomena from specific, simple rules [1]. In the context of computer science, there are CA algorithms—both one‐dimensional (1D) and two‐dimensional (2D)—that have been found to be Turing complete (computationally universal) [1]. Researchers have historically proposed variations of 2D CA as novel parallel computational architectures, as each cell in a 2D CA grid may be updated independently, and therefore in parallel [2]. In the natural sciences, CA offers computationally efficient frameworks for simulating emergent/coarse‐grained dynamics in physical systems across a broad spectrum of lengthscales, timescales, and substrates [1, 3, 4, 5, 6].

An intriguing feature of CA in interdisciplinary contexts is the very quality that makes them challenging to formally characterize. Namely, this is the fact that CA often exhibit highly complex or chaotic, “computationally irreducible” behavior, implying that any prediction of the state S of a CA system n timesteps in the future must take at least n discrete computational steps to produce—there are no lossless “shortcuts” or analytical representations of the system as a function of n and S [7, 8, 9]. Thus, while CA systems have served well as pedagogical tools for qualitatively illustrating the emergence of complexity from simple underlying rules, they remain widely unutilized as quantitative predictive models for physical systems. With regard to this, Springer and Kenyon (2020) [10] showed that it often takes much larger convolutional neural networks (CNNs) than theoretically necessary to reliably learn the state‐transition rules of the 2D CA system “Conway's Game of Life” (*Life*) [11]. Results such as these have led to the common assumption that it is unlikely that AI will be able to perfectly predict the nth state of a particular CA system based on an initial condition (IC).

However, even if a given CA system is likely irreducible, there is nothing stopping us from slowly evolving state transition rules to generate some target emergent behavior. Neural cellular automata [12] (NCA) have also emerged as a subfield focused on synthesizing the tunability of neural networks with the collective dynamics of cellular automata. NCA have been shown to enable dynamical CA systems to generate emergent visual patterns with tunable steady states [13]. The key attribute of NCA is that each cell in a 2D grid is encoded as a vector and the global update rule is itself a neural network (CNN [12], Attention Layer [14], etc.) which intakes cells' local neighborhood cell vectors and outputs incremental updates to cells' states. Using this framework, the update rule can be tuned iteratively through backpropagation. While this field shows real promise for the future of CA‐based dynamic mimicry of synthetic and real‐world systems, the fact that the state transition rules in NCA are themselves neural networks fundamentally limits their *interpretability*. In essence, we cannot easily “peer into” these systems to find what leads to their unique, dynamical evolution—their state transition rules are, in effect, “black boxes.”

Still, one does not need to use neural networks as update rules to develop a framework for iterative tuning of emergent behavior. In the case of spacetime‐inhomogeneous cellular automata [15], randomly “mutating” the placement of CA rules within “spacetime” and keeping only those mutations which advance a particular performance metric while rejecting the rest has been shown to eventually lead to a configuration engendering a desired behavior; though, this strategy is undoubtedly under‐researched and may turn out to be limited depending on the particular performance metric, rule space, and grid size.

On the other side of the computer science discipline, transformer models have gained widespread popularity across both computer science and the natural sciences for their domain‐agnostic learning capabilities and favorable scaling behaviors [16, 17], including but not limited to genomic analysis [18], meteorology and climate prediction [19, 20, 21], protein structure prediction [22], strategic decision‐making in games [23, 24, 25], computational linguistics and text generation [26], audio and speech recognition [27, 28, 29], computer vision and image analysis [30], and materials science [31, 32, 33, 34], among others.

We have recently shown [35] that generative pretrained transformer models (GPTs) are capable of learning the state‐transition rules of CA from raw data. In the resulting model, LifeGPT, we showed that a decoder‐only transformer model was able to learn to apply the update rules of *Life* on a 32×32 toroidal grid, with no prior knowledge of grid topology, presenting near‐perfect accuracy. Our prior [35] work also demonstrated that transformer models generalize better over CA datasets when the distribution of ICs varies in entropy/ordering, as opposed to being entirely high‐entropy/disordered. Following this, Burtsev (2024) [36] used elementary (1D) CA to investigate the ability for GPTs to predict long‐term behavior in complex systems, notably finding that increasing model depth, including intermediate future states, and explicitly including CA ruleset inference into training loss improved performance. These results support the hypothesis we presented in our previous work [35] that CA rules could function as an internal “world model,” as posited by Ha and Schmidhuber (2018) [37]: a simplified construct of the system at hand that serves to guide the model in the “correct direction.”

Similarly meshing transformer models with artificial life, Kumar et al. (2024) proposed Automated Search for Artificial Life (ASAL) [38], a paradigm incorporating vision‐language foundation models (FMs) for discovering and taxonomizing artificial life (ALife) simulations across multiple substrates (Boids [39], Particle Life [40, 41], Life‐like CA [42, 43], Lenia [44, 45], and NCA [12]). In this work, vision language FMs were used within a larger genetic algorithm to generate representations of ALife simulations (spacetimes) in order to match natural language prompts, maximize simulation “open‐endedness,” and maximize simulation novelty to explore/taxonomize simulation‐space.

Both Kumar et al. [38] and Burtsev [36] showed that training transformer models on large sets of rules leads to adequate model generalization for emergent dynamics in ALife systems. However, Burtsev focused on 1D CA forecasting while Kumar et al. focused on exploration and taxonomy of “simulation space” for a variety of 2D substrates, including Life‐like CA. Neither work investigated forecasting the evolution of 2D CA, nor did they investigate whether rules could be inferred/extracted by an AI system, given some input ALife time‐evolution/CA orbit.

**FIGURE 2:**
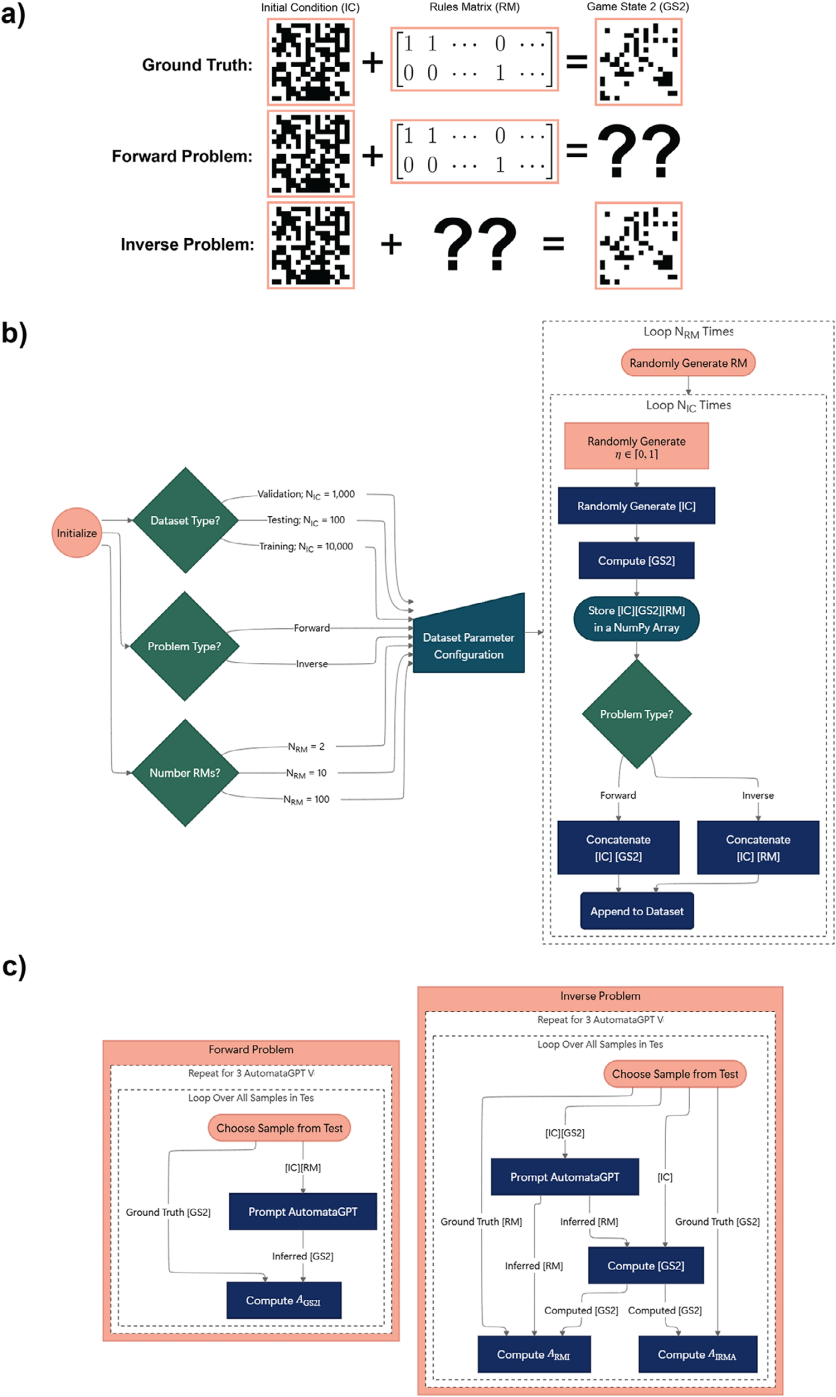
(a) Illustration of the forward and inverse problems investigated in this study, with respect to the ground truth. The ground truth shows the standard relationship between the IC, RM, and GS2 for a given CA system sample: the rules defined by the RM are applied to the IC to compute GS2. Question marks represent the unknowns (GS2 or RM) that AutomataGPT is tasked with inferring for each problem. (b) Dataset generation procedure for datasets used to train, validate, and test AutomataGPT. For each $N_{\text{RM}}$ value, a unique version of AutomataGPT was trained from scratch —three models per problem for a total of six unique versions of AutomataGPT (see Table 2). It should be noted that in addition to the training, validation, and testing sets generated for each AutomataGPT version, one additional testing set ($N_{\text{RM}}=100;N_{\text{IC}}=2$) was generated for characterizing performance *across all* AutomataGPT versions. (c) Experimental procedure for characterizing the performance of versions of AutomataGPT for both the forward and inverse problems (using the same $N_{\text{RM}}=100;N_{\text{IC}}=2$ testing set).

This prior work leaves open the following questions: (1) Does the improved *forecasting* (Figure 2a) performance of GPTs enabled by explicitly encoding rules in training data (as shown by Burtsev) extend to a setting for systems with increased dimensionalities (2D) and topological complexities (toroidal grid)? (2) How well can GPTs solve an inverse problem (Figure 2a) in which successive states of a CA system are given and the model is tasked with *inferring rules* that fit the system's dynamics?

We believe answering these questions is critical to the development of novel, CA‐based, physical simulation techniques. Considering that morphological development in multicellular organisms is governed by the global emergent dynamics of local cellular interactions [46, 47], generating CA rulesets representative of these biological systems using GPTs prompted with empirical data could be an effective technique for forecasting time‐evolution, which would be highly beneficial for fields such as biology and tissue engineering [48, 49, 50, 51, 52, 53]. Furthermore, many nonbiological natural phenomena are emergent effects stemming from the interactions of many particles or otherwise discrete units of matter [54, 55, 56, 57]. To this end, generating CA rules from empirical data might help us bridge the explanatory gap between length scales across many systems and scientific domains.

CA span a broad design space distinguished by lattice geometry and dimensionality, neighborhood choice (e.g., von Neumann vs. Moore [58] in 2D), state alphabets, boundary conditions, update synchrony, and whether rules are uniform or vary across sites; see Bhattacharjee et al. (2020) [59] for a comprehensive taxonomy and applications overview. In this work, we deliberately focus on a narrow, well‐defined subset: two‐dimensional, binary, deterministic, isotropic (count‐based, nondirectional) rules on a square lattice with a radius‐1 (r=1) Moore neighborhood and periodic (toroidal) boundary conditions (updates depend only on a cell's state and its neighbor count). Nonuniform and hybrid CA, where local rules or neighborhoods differ across sites, exhibit distinct behaviors and remain outside our present scope. This restriction provides a controlled testbed for the questions we aim to answer, while situating this work within a well‐studied corner of the broader CA landscape. A crucial consequence of this restriction is that the neighborhood radius is treated here as a fixed modeling choice rather than a quantity inferred from data. In the present study, all training, inference, and validation are performed within a radius‐1 Moore‐neighborhood surrogate class. Accordingly, our inverse problem is not joint discovery of the correct CA family and radius, but rule inference *within* a specified local hypothesis class. This distinction is important for interpreting all subsequent claims, especially when discussing extensions to non‐CA systems.

Thus, we propose *AutomataGPT*, a decoder‐only GPT model that we trained in two distinct manners to (1) apply update rules to 2D binary deterministic non‐directional CA with a r=1 Moore neighborhood [58] and (2) infer CA rules given an initial condition (IC) and a subsequent game state (GS2). For each problem, we train three versions of AutomataGPT from scratch on different sets of CA rules (different numbers of RMs: $N_{\text{RM}}\in \left\{2,10,100\right\}$) and on both the forward and inverse problems (Figure 2a). Our experiments investigate how the size of the rule space (the number of RMs used to construct a CA dataset—see Figure 2b) used for training impacts accuracy for both problems (Figure 2c), with a focus on RM inference performance as a function of test sample RM dissimilarity relative to RMs in training data. In doing so, we benchmark generalization capabilities and inductive bias across rule space for different AutomataGPT versions. We did *not* investigate AutomataGPT's sensitivity to different IC distributions during training, as our previous LifeGPT [35] study showed that, for the forward problem in *Life*, broad‐entropy IC distributions were sufficient to enable model generalization across diverse ICs (including both stochastically generated ICs and those generated by iterating CA systems).

In this sense, the present work should be viewed as a controlled benchmark of symbolic rule inference under strong structural priors: locality, determinism, isotropy, binary state space, and fixed radius. The question addressed here is therefore not whether arbitrary dynamical systems can be uniquely inverted into CA rules, but whether transformer models can recover and apply explicit local rules reliably when inference is restricted to a fixed radius‐1 binary deterministic surrogate class. This restricted setting also permits a sharper theoretical treatment of the inverse problem; in the Supporting Information, we formalize how fixed surrogate neighborhood class, pairwise sampling, and neighborhood coverage jointly determine what can and cannot be inferred from IC–GS2 data.

What we define as the inverse problem may also be thought of as a longstanding challenge in the CA literature known as the global‐to‐local mapping problem [60]: recover a local update rule that reproduces observed global behavior or specified macroscopic properties. Classical efforts range from direct attempts to engineer attractor basins for desired dynamics (e.g., Wuensche; Askenazi) to the much more influential line based on evolutionary computation, including early work by Packard and Koza and the Santa Fe “EVCA” program of Mitchell and colleagues on density classification and synchronization, where genetic algorithms and simulated annealing evolve rule populations toward task performance [61, 62, 63, 64, 65]. Subsequent refinements introduced co‐evolution of rules and initial conditions to mitigate sampling bias [66, 67, 68], and explored hybrid/nonuniform CA (different local rules across sites) to capture more complex computations, albeit at the cost of a vastly enlarged search space; cellular programming and related schemes address this scaling [69]. Parallel work constrained evolution to analytically tractable subclasses (additive/linear or group CA), reducing the hypothesis space and improving convergence [70, 71]. The core difficulty is combinatorial: the set of possible global behaviors dwarfs the set of admissible local rules, so unconstrained inversion is hard, and success typically hinges on synthetic data, task structure, or strong priors on rule classes. Our approach differs in spirit: rather than search per‐instance, we amortize the inverse problem by training a decoder‐only transformer on many synthetic CA trajectories within a controlled, isotropic, binary Moore‐neighborhood family. This yields a model that can generate rules and forecast states for unseen rules without an explicit evolutionary loop, retaining a clear path to move beyond synthetic settings. We emphasize that our study does not attempt to resolve the general global‐to‐local mapping problem, which is theoretically intractable in unconstrained settings. Without strong structural priors, it is impossible to uniquely identify a local rule from arbitrary global trajectories. Instead, we explicitly restrict ourselves to synthetic 2D CA with local, non‐directional (isotropic) radius‐1 rules, so AutomataGPT does not need to extensively search the entire global mapping space. AutomataGPT is therefore designed for systems where local causal structure is assumed a priori and represented within a fixed surrogate neighborhood class, providing a controlled benchmark to test whether transformer models can recover rule matrices and forecast dynamics under these constraints. In particular, the present study does not attempt to infer the appropriate neighborhood radius from data, nor to establish that a local CA surrogate exists for an arbitrary system. A corresponding conditional theoretical framework is developed in the Supporting Information, where we formalize pairwise identifiability, the role of neighborhood coverage, and the interpretation of coarse‐grained non‐CA dynamics within a fixed surrogate CA class.

While this study focuses on demonstrating the feasibility of ruleset inference and forecasting within 2D CA systems, we view our work as a novel neuro‐symbolic approach [72] in which neural models are used to infer explicit, executable symbolic rules from data. Prior efforts related to rule or program inference have included neural program induction and synthesis [73, 74, 75], symbolic regression and equation discovery [76, 77, 78], evolutionary and genetic searches over CA rule spaces [79, 80, 81], and neural or transformer‐based models trained to forecast dynamical systems without explicit rule recovery [10, 35, 36]. In contrast to these approaches, AutomataGPT amortizes the discovery of local update rules directly from spatiotemporal observations, yielding symbolic rulesets that can be executed, inspected, and tested. Distinct from imposing symbolic structure a priori or reasoning over fixed knowledge bases [34, 82, 83], this coupling of neural learning with symbolic rule extraction enables data‐driven identification of executable local rule surrogates, and may provide interpretable hypotheses about governing principles in settings where locality is a reasonable modeling prior. The broader implications of this work extend to real‐world applications in biology, physics, and other natural sciences. These applications, though largely beyond the scope of this paper, highlight the potential for CA‐based approaches to bridge explanatory gaps across scales and domains.

### 1.1 Paper Outline

We begin by investigating versions of AutomataGPT trained on the forward problem. We define an accuracy metric, interpret data from experiments testing three versions trained on different training sets, and offer questions for future research. We then investigate versions of AutomataGPT trained on the inverse problem with distinct training data, defining two new accuracy metrics and providing data from our experiments. We then interpret our findings, commenting on the relationship between training data and AutomataGPT's generalization capabilities, inductive bias, and creativity. We go on to discuss limitations regarding the inverse problem, including the dependence of inference on locality assumptions, neighborhood coverage, and the fixed surrogate rule class used throughout this study, and conclude by discussing implications for modeling real‐world dynamical systems and potential next steps.

## 2 Results and Discussion

### 2.1 Forward Problem

We defined the forward problem as: *“If given a flattened representation of the RM and an IC, can AutomataGPT accurately generate the following state of the CA grid in accordance with the RM and IC, ‘game state 2’ (GS2)?”* (Figure 1).

**FIGURE 3:**
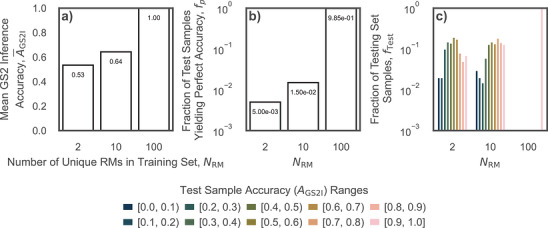
Accuracy increases significantly with more extensive training on rulesets, demonstrating AutomataGPT's generalization from 2 to 100 rules. (a) Bar chart illustrating the change in AutomataGPT's accuracy on the forward problem averaged over the entire testing set (${\bar{A}}_{\text{GS2I}}$) versus the number of RMs used for training ($N_{\text{RM}}$). (b) Bar chart depicting the effect of $N_{\text{RM}}$ on the fraction of perfect GS2 inferences across the testing set ($f_{p}$). (c) $A_{\text{GS2I}}$ histogram for models trained with varying $N_{\text{RM}}$, where $f_{\text{Test}}$ is the fraction of samples in the testing set. The grayscale legend at the bottom of the figure denotes $A_{\text{GS2I}}$ ranges for the histograms. Charts *(a–c)* all indicate that, in the case of the forward problem, as AutomataGPT was trained on larger rule spaces, its inference accuracy consistently increased, reaching near‐perfect accuracy for $N_{\text{RM}}=100$. This suggests that for $N_{\text{RM}}$, AutomataGPT was effectively trained to be a CA computer, capable of applying any RM (corresponding to a 2D binary deterministic automaton) to any 16×16 binary IC to generate a near‐perfect GS2 inference. Statistical details: Data are reported as mean accuracy values with n=200 test cases per model; error fractions ($f_{e}$) and perfect fractions ($f_{p}$) were computed directly from the distributions, and histogram frequencies sum to 1 across the ten accuracy bins.

Figure 3 illustrates the change in AutomataGPT's inference accuracy for the forward problem as the model is trained from scratch on an increasing number of RMs. For each version of AutomataGPT, we quantify forecasting accuracy using the accuracy of GS2 inference ($A_{\text{GS2I}}$), where $A_{\text{GS2I}}$ is calculated independently for each sample in the testing set, as is defined as follows:

(1) $$A_{\text{GS2I}}=\frac{\gamma_{c}}{\gamma_{t}}$$

where $\gamma_{c}$ is the number of correctly *inferred* GS2 tokens and $\gamma_{t}=16^{2}=256$ is the total number of tokens comprising each GS2. Furthermore, ${\bar{A}}_{\text{GS2I}}$ is the average accuracy of GS2 inference (averaged across all samples in the testing set):

(2) $${\bar{A}}_{\text{GS2I}}=\frac{1}{N_{S}}\sum_{i=0}^{N_{S}-1}A_{\text{GS2I},i},$$

where $A_{\text{GS2I},i}$ is the accuracy of GS2 inference for the ith sample in the testing set and $N_{S}$ is the number of samples in the testing set ($N_{S}=200$).

We found that ${\bar{A}}_{\text{GS2I}}$ increased as AutomataGPT was trained on increasing $N_{\text{RM}}$ (Figure 3a). This suggests that training GPTs on larger sections of rule space improves their ability to become generally programmable for running CA. In other words, when a GPT model is trained on many examples of CA governed only by a *few* distinct RMs, it likely finds nonintuitive token relationships that do not generalize for unseen RMs; however, when a GPT model is trained on many examples of CA with *many* corresponding RMs, it successfully learns a “near‐correct” method of applying the inputted RM onto the inputted IC to yield a GS2, becoming *rules‐agnostic*. Still, in our experiment for $N_{\text{RM}}=100$, the model was not perfect, yielding perfect GS2 inferences 98.5% of the time, inferring 3 out of 200 GS2s imperfectly (Figure 3b). Nevertheless, the difference is stark—*the* $N_{\text{RM}}=100$ *AutomataGPT version is near‐perfect on the forward problem after being trained on less than* 0.04% *of all possible rules* (see Section 4.8 and Equation 12). $N_{\text{RM}}=100$ was trained using 1e4 ICs per RM, coming out to one million “games” in the training set. (We can safely assume that the vast majority of these games have unique ICs, due to the massive IC configuration space: $2^{16\times 16}\approx 1.15e77$ binary ICs for a 16×16 grid. This means that the $N_{\text{RM}}=100$ AutomataGPT version was trained on only $100\%\times \frac{1e6}{1.15e77}\approx 8.64\times 10^{-70}\%$ of all possible ICs.) This demonstrates the incredible ability of *small* (relative to the state‐of‐the‐art LLMs) GPTs to generalize without ultra‐large training sets.

Given these results, we suspect that the observed increase in inference accuracy would continue to approach perfection if $N_{\text{RM}}$ were further increased. Furthermore, it is shown in Figure 3c that for $N_{\text{RM}}=100$, 100% of samples tested yielded $A_{\text{GS2I}}\geq 0.9$ (≥90% forecasting accuracy). These results exist in contrast to the relatively flat accuracy distributions for $N_{\text{RM}}=2$ and $N_{\text{RM}}=10$. Notably, $N_{\text{RM}}=10$ did show some minor improvement over $N_{\text{RM}}=2$, though it remains unclear precisely at what $N_{\text{RM}}$ AutomataGPT might begin to improve rapidly, or whether the model undergoes a “phase transition” at a critical $N_{\text{RM}}$. Future work is warranted, as we consider this question to be outside the scope of the present study.

These results suggest that *it is generally possible for a decoder‐only transformer model to be trained such that it becomes a near‐perfect, generally‐programmable, 2D binary deterministic CA computer, and that increasing the breadth of the rules in its training data will improve its single‐timestep forecasting accuracy, even if the number of rules it is trained on only comprises a small fraction of the entire rule space*. Still, it is difficult to say with much certainty at what $N_{\text{RM}}$, if any, AutomataGPT would reach 100% forecasting accuracy—it may be that GPTs will often get stuck in local minima instead of effectively generalizing for this type of problem. Further research is required to address these questions. Moreover, while this work focuses on one‐step prediction as a proof of concept, future work is needed to benchmark long‐horizons and extend to richer CA (multi‐state, directional, stochastic) as well as coarse‐grained/global targets.

### 2.2 Inverse Problem

We defined the inverse problem as: *“Given an IC and GS2 for a CA system, can AutomataGPT accurately infer the RM?”* (Figure 1.)

**FIGURE 4:**
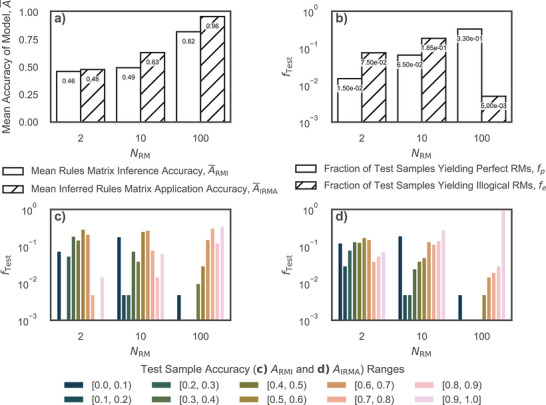
(a) Bar chart illustrating the change in inverse problem inference accuracy averaged over the testing set ($\bar{A}$) for two distinct accuracy metrics (Rules Matrix Inference Accuracy—${\bar{A}}_{\text{RMI}}$—and Inferred Rules Matrix Application Accuracy—${\bar{A}}_{\text{IRMA}}$) versus the number of RMs used for training ($N_{\text{RM}}$). This chart shows that as AutomataGPT is trained on a larger rule space, its inferences become more accurate overall. That fact that ${\bar{A}}_{\text{RMI}}<{\bar{A}}_{\text{IRMA}}\forall N_{\text{RM}}$ suggests that even relatively inaccurate RM inferences can yield somewhat more accurate GS2 computations when applied a given sample's IC. Moreover, the widening gap between ${\bar{A}}_{\text{RMI}}$ and ${\bar{A}}_{\text{IRMA}}$ as $N_{\text{RM}}$ increases suggests that training on larger rule spaces enables AutomataGPT to infer more accurate *degenerate solutions* (RMs that are not the same as ground truth but still yield somewhat accurate GS2s when applied to ICs). (b) Bar chart depicting the effect of $N_{\text{RM}}$ on the fraction of perfect RM inferences across the testing set ($f_{p}$) and the fraction of errors (inferred RMs that are illogical) across the testing set ($f_{e}$). It is observed that training on a larger rule space enables fewer illogical RM inferences and more perfect RM inferences. (c) $A_{\text{RMI}}$ histogram for models trained with varying $N_{\text{RM}}$. (d) $A_{\text{IRMA}}$ histogram for models trained with varying $N_{\text{RM}}$. Charts (c) and (d) indicate the same trends as (a) but in more detail, showing the fraction of individual test samples belonging to each A range. Observing these data, it is clear that the improvement gains brought on by training on a larger rule space are more substantial when considering degenerate solutions. One can interpret these findings as evidence of the model becoming more “creative” as it is trained on larger rule spaces. Statistical details: n=200 test cases were evaluated for each model; bar plots report mean accuracies and fractions ($f_{p}$, $f_{e}$), while histogram panels show relative frequency of accuracies per bin, normalized to the total number of samples.

**FIGURE 5:**
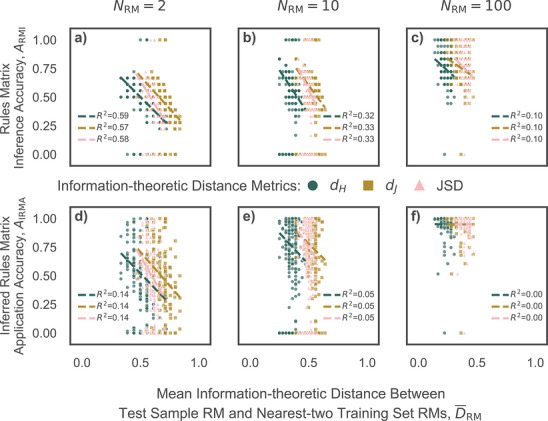
Plots depicting the capabilities of varying versions of AutomataGPT to infer RMs (the inverse problem) for a testing set with samples generated using RMs not present in any training data. Specifically, model accuracy vs. RM difference averaged across the closest two RMs in the training set (${\bar{D}}_{\text{RM}}$) is plotted for varying difference metrics, models, and accuracy metrics. The ${\bar{D}}_{\text{RM}}$ metrics used were Hamming distance ($d_{H}$), Jaccard distance ($d_{J}$), and Jensen–Shannon Divergence (JSD). (a) Rules Matrix Inference Accuracy ($A_{\text{RMI}}$) versus ${\bar{D}}_{\text{RM}}$ for $N_{\text{RM}}=2$. (b) $A_{\text{RMI}}$ versus ${\bar{D}}_{\text{RM}}$ for $N_{\text{RM}}=10$. (c) $A_{\text{RMI}}$ versus ${\bar{D}}_{\text{RM}}$ for $N_{\text{RM}}=100$. (d) Inferred Rules Matrix Application Accuracy ($A_{\text{IRMA}}$) versus (${\bar{D}}_{\text{RM}}$) for $N_{\text{RM}}=2$. (e) $A_{\text{IRMA}}$ versus ${\bar{D}}_{\text{RM}}$ for $N_{\text{RM}}=10$. (f) $A_{\text{IRMA}}$ versus ${\bar{D}}_{\text{RM}}$ for $N_{\text{RM}}=100$. The data shown across plots (a–f) provide insight into the inductive bias of AutomataGPT versions depending on the number of RMs used for training. In particular, a decreasing $R^{2}$ value across all ${\bar{D}}_{\text{RM}}s$ as $N_{\text{RM}}$ increases indicates that as AutomataGPT learns from larger rule spaces, it more effectively generalizes across rules it has never seen before, with the rules that is *has* seen playing an increasingly insignificant role in shaping its responses. Furthermore, the fact that, for $N_{\text{RM}}=100$, $R^{2}=0.00$ for $A_{\text{IRMA}}$ while $R^{2}=0.10$ for $A_{\text{RMI}}$ suggests that this AutomataGPT version is more likely to generate degenerate solutions for the inverse problem when a given test sample has been generated with an RM very different from RMs in the model's training set (creativity is prompted by “unusual systems”)—notably, such degenerate solutions are just as likely to reflect the dynamics of the test sample system as non‐degenerate solutions. Note: Accuracies of 0 and 1 were excluded from the regressions, ensuring that regressions only correlated the accuracies of *imperfect‐but‐still‐logical* RM inferences with ${\bar{D}}_{\text{RM}}$. Statistical details: Scatter plots include all test cases (n=200 per model), but ordinary least‐squares regression fits exclude boundary accuracies of 0 and 1; reported $R^{2}$ values therefore reflect correlations between ${\bar{D}}_{\text{RM}}$ and imperfect‐but‐logical inferences only.

Figures 4 and 5 illustrate the change in AutomataGPT's inference accuracy for the inverse problem as it was repeatedly trained from scratch on an increasing number of RMs. For each version of AutomataGPT, we quantify inference accuracy using *two* metrics.

The first accuracy metric is $A_{\text{RMI}}$ (Rules Matrix Inference Accuracy), defined as:

(3) $$A_{\text{RMI}}=\left\{\begin{array}{cc} 1, & \text{if the inferred RM yields the exact ground truth GS2}\\ 0, & \text{if the inferred RM is illogical}\\ \frac{\rho_{c}}{\rho_{t}}, & \text{otherwise} \end{array}\right.$$

where $\rho_{c}$ is the number of correctly inferred tokens in the RM, and $\rho_{t}=36$ is the total number of tokens comprising each RM. $A_{\text{RMI}}$ was defined this way in order to not penalize our model for finding alternative RMs equally capable of *perfectly* describing the CA system in the testing set.

The second accuracy metric is $A_{\text{IRMA}}$ (Inferred Rules Matrix Application Accuracy), defined as

(4) $$A_{\text{IRMA}}=\left\{\begin{array}{cc} 0, & \text{if the inferred RM is illogical}\\ \frac{\gamma_{c}}{\gamma_{t}}, & \text{otherwise} \end{array}\right.$$

where $\gamma_{c}$ is the number of correctly computed tokens for GS2 when *applying* the inferred RM to the IC, and $\gamma_{t}=16^{2}=256$ is the total number of tokens comprising each GS2. $A_{\text{IRMA}}$ captures the ability of our model to infer imperfect RMs that still reasonably capture the behavior of the CA system in the testing set.

We also considered the average values of these two accuracy metrics, across all samples in the testing set

(5) $${\bar{A}}_{\text{RMI}}=\frac{1}{N_{S}}\sum_{i=0}^{N_{S}-1}A_{\text{RMI},i},$$

(6) $${\bar{A}}_{\text{IRMA}}=\frac{1}{N_{S}}\sum_{i=0}^{N_{S}-1}A_{\text{IRMA},i},$$

where $A_{\text{RMI},i}$ and $A_{\text{IRMA},i}$ were the accuracies for the ith samples in the testing set and $N_{S}$ was the number of samples in the testing set ($N_{S}=200$).

We found that for both accuracy metrics, the average accuracy ($\bar{A}$) increased as AutomataGPT was trained with higher $N_{\text{RM}}$ (Figure 4a). This is evidence that increasing the number of RMs in the training set results in an increased ability for our model to generalize across rule space. The fraction of samples for which AutomataGPT perfectly inferred RMs (including inferring degenerate RMs) also steadily increased with $N_{\text{RM}}$(Figure 4b).

Also, for ${\bar{A}}_{\text{RMI}}$, the fraction of samples in the testing set leading to the inference of an illogical RM (where probabilities of state transition for a given metastate do not add to 1), which we termed the “error fraction” ($f_{e}$), initially increased from $N_{\text{RM}}=2$ to (Figure 1), but decreased by over an order of magnitude from $N_{\text{RM}}=10$ to $N_{\text{RM}}=100$ (Figure 4b). This suggests that for a sufficiently great number of RMs AutomataGPT learns to infer mostly *logical* RMs. We suspect that for even larger $N_{\text{RM}}s$, this effect would be intensified, and AutomataGPT's illogical inferences would become exceedingly rare. Another indication that increasing the number of RMs was beneficial overall to model performance was that AutomataGPT's accuracy distribution improved favorably with higher $N_{\text{RM}}$ when considering both $A_{\text{RMI}}$ and $A_{\text{IRMA}}$ (Figure 4c).

While these results initially lent hope to the notion that a large enough training set might allow AutomataGPT to accurately infer rules for *any* 2D binary deterministic CA system, there was still the possibility that the diversity of the RMs in AutomataGPT's training data had inevitably limited its inference capabilities. It seemed hypothetically feasible that samples in the testing set corresponding to CA systems that were computed with RMs more similar to those used to create the training set would always result in more accurate RM inferences. *If* this was found to be true, then it would be an indication of significant inductive bias.

To test for this possibility, we defined ${\bar{D}}_{\text{RM}}$, representing an information‐theoretic distance quantifying the average dissimilarity between the testing sample's RM and the “closest” *two* RMs in AutomataGPT's training set. While we refer to ${\bar{D}}_{\text{RM}}$ generally, we specifically utilized three established dissimilarity metrics in order to remove metric‐induced bias from our results: (1) *Hamming distance*—$d_{H}$ [84]; (2) *Jaccard distance* (or one minus the *Jaccard index*, which is also known as the *Tanimoto coefficient*)—$d_{J}$ [85, 86]; (3) *Jensen–Shannon divergence* (also known as the *information radius*)—JSD [87].

As AutomataGPT was trained on an increasing number of rulesets, inverse problem accuracies (both $A_{\text{RMI}}$ and $A_{\text{IRMA}}$) became less correlated with ${\bar{D}}_{\text{RM}}$. This was shown by the linear regressions in Figure 5, which approximated the function ${\bar{A}}_{\text{RMI}}\left({\bar{D}}_{\text{RM}}\right)$ $\forall {\bar{A}}_{\text{RMI}}\notin \left\{0,1\right\}$ in Figure 5a–c and ${\bar{A}}_{\text{IRMA}}\left({\bar{D}}_{\text{RM}}\right)$ $\forall {\bar{A}}_{\text{IRMA}}\notin \left\{0,1\right\}$ in Figure 5d–f. Accuracies of 0 and 1 were excluded from the regressions since the former reflected illogically inferred RMs (not interpretable as adjacency matrices) and the latter reflected perfectly inferred RMs (matching ground truth).

This trend held true across all ${\bar{D}}_{\text{RM}}s$ ($d_{H}$, $d_{J}$, and JSD). This suggested that *as versions of AutomataGPT were trained on increasing numbers of unique rulesets, they became increasingly general*—the rules governing the test sample system became less correlated with inference accuracy.

Furthermore, the $R^{2}$ values across all ${\bar{D}}_{\text{RM}}s$ decreased for all models when switching the chosen accuracy metric from $A_{\text{RMI}}$ to $A_{\text{IRMA}}$ (Figure 5). In particular, for $N_{\text{RM}}=100$, $R^{2}$ dropped from 0.10 to 0.00 (Figure 5c,f), which showed that while AutomataGPT was more likely to infer RMs close to ground truth for smaller ${\bar{D}}_{\text{RM}}$, the extent to which these inferred RMs were accurate reflections of the underlying dynamics of the test sample system was not correlated with ${\bar{D}}_{\text{RM}}$. Fundamentally, this means that *AutomataGPT is more likely to come up with “creative” solutions to the inverse problem (non‐ground‐truth RM inference) if the test sample RM was far from all RMs in its training set*. Importantly, for $N_{\text{RM}}=100$, AutomataGPT was just as likely to accurately infer RMs that reliably reproduced GS2 for systems with low ${\bar{D}}_{\text{RM}}$ as for those with high ${\bar{D}}_{\text{RM}}$ (Figure 5f)—*creativity does not trade‐off with accuracy, and AutomataGPT's best version exhibits virtually no inductive bias in this regard*.

A central caveat is that with a single timestep (IC→GS2), many metastates may be unobserved, leaving the corresponding columns of the RM unconstrained. Multiple rulesets can therefore yield the same successor state; that is, degeneracy is induced by the data, not by the model. If this is the case, then if given more time‐evolution data (longer orbits) for CA systems, we might expect AutomataGPT to become better at inferring ground‐truth RMs, as the space of degenerate solutions would shrink. (We discuss this limitation further in the next section and in Section S1.) Another related question is: “How does Automata GPT's *sampling temperature* affect creativity and accuracy with regard to the inverse problem?” We deem these inquiries to be beyond the scope of this paper and worthy of further research.

Finally, we investigated how perfect ($A_{\text{RMI}}=1;A_{\text{IRMA}}=1$) degenerate RMs may be used to compute long orbits—how long it takes for these orbits to diverge from ground truth. We define a new metric we call the *strict* rules matrix inference accuracy

(7) $$A_{\text{RMI, strict}}=\frac{\rho_{c}}{\rho_{t}},$$

and the in‐orbit rules matrix application accuracy for a given game state g:

(8) $$A_{\text{IRMA}}\left(g\right)=\frac{\gamma_{c,\text{GS}g}}{\gamma_{t}},$$

where $\gamma_{c,\text{GS}g}$ is the number of tokens correctly predicted in GSg. The first metric, $A_{\text{RMI}}$, measures the direct agreement between inferred and ground‐truth rules matrices. The second, $A_{\text{IRMA}}\left(g\right)$, evaluates how well the inferred rules reproduce a specific state in the orbit when applied recursively from the IC.

**FIGURE 6:**
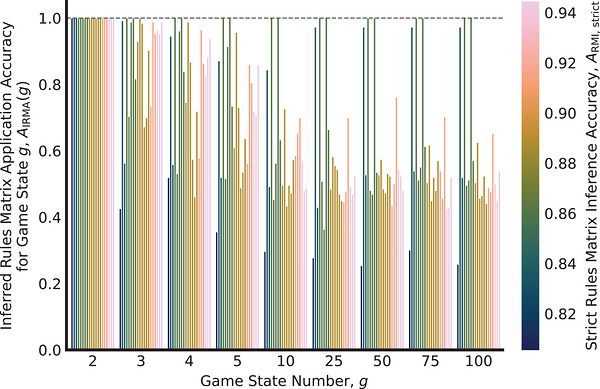
Bar plot showing how the accuracy of inferred rules matrix application for a given game state, $A_{\text{IRMA}}\left(g\right)$, varies with the game state, g, where GS2 corresponds to g=2, for long orbits across many testing samples. The data reflect the use of perfect degenerate solution RMs—extracted by AutomataGPT ($N_{\text{RM}}=100$) from random testing set samples and confirmed to yield 100% accurate GS2s—to recursively compute orbits from ICs for each sample. These orbits were then compared to the ground truth orbits to compute $A_{\text{IRMA}}\left(g\right)$ for each chosen game state (g). Samples were purposefully chosen to include only those for which $A_{\text{IRMA}}\left(2\right)=1$, so as to limit this investigation to only perfect degenerate solution RMs. Samples are colored based the strict accuracy of rules matrix inference, $A_{\text{RMI, strict}}$. These data suggest that most perfect degenerate solution RMs inferred by AutomataGPT ($N_{\text{RM}}=100$) are only consistently accurate for GS2; orbits usually sharply diverge from ground truth beginning at g=3. There are a few outliers, including two samples where orbits did not diverge at all, and one where accuracy initially decreased and later increased. There does not appear to be a significant correlation between $A_{\text{RMI, strict}}$ and $A_{\text{IRMA}}\left(g\right)$ for any g. Most orbits settle around $A_{\text{IRMA}}\left(g\right)\approx 0.5$ by g=10. Statistical details: Of the 50 samples tested, 20 qualified for analysis by exhibiting perfect GS2 accuracy ($A_{\text{IRMA}}\left(2\right)=1$); for these, orbit divergence was tracked over 100 timesteps, with $A_{\text{IRMA}}\left(g\right)$ computed per sample and colored by strict rules matrix inference accuracy $A_{\text{RMI, strict}}$.

Figure 6 shows $A_{\text{IRMA}}\left(g\right)$ for each chosen game state g (g=2 corresponds to GS2) across many testing samples. Perfect degenerate solution RMs, extracted by AutomataGPT ($N_{\text{RM}}=100$) from random testing set samples and confirmed to yield 100% accurate GS2s, were recursively applied from the IC to generate orbits, which were then compared to the ground truth. By construction, $A_{\text{IRMA}}\left(2\right)=1$ for all samples, allowing us to focus on divergence beyond GS2. The results indicate that most perfect degenerate solution RMs are only consistently accurate for GS2, with accuracy typically dropping sharply at g=3 and stabilizing near $A_{\text{IRMA}}\left(g\right)\approx 0.5$ by g=10. A few outliers maintain high accuracy throughout the orbit, and there is no apparent correlation between $A_{\text{RMI, strict}}$ and $A_{\text{IRMA}}\left(g\right)$ for any g. *This behavior highlights a key limitation: even rules matrices that are perfect under the current evaluation criteria may fail to generalize beyond the immediate successor state*. This was likely due to one or more metastates being absent from an IC in a given testing sample, so AutomataGPT lacked the information necessary to determine which state transitions those missing metastates should be assigned in the RM. Though we did not explicitly test imperfectly inferred RMs, we reasonably conclude from our results that imperfectly inferred RMs would fair even worse than perfect degenerate solution RMs, since they do not even reproduce GS2 reliably which significantly perturbs their respective orbits. In practice, this suggests that a future version of AutomataGPT should be trained and evaluated on longer orbits (since long orbits decrease the odds of missing metastates) to discourage degenerate solutions, increase $A_{\text{RMI, strict}}$, and improve the stability of inferred dynamics over extended trajectories; this is out of the scope of this work but is a key future research direction.

### 2.3 Limitations and Challenges in Solving the Inverse Problem

**FIGURE 7:**
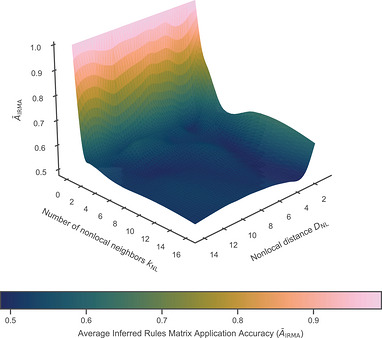
Smoothed surface plot showing the average inferred rules matrix application accuracy (${\bar{A}}_{\mathrm{IRMA}}$) as a function of the number of nonlocal neighbors $k_{\mathrm{NL}}$ and their Chebyshev distance $D_{\mathrm{NL}}$. As nonlocal interactions are introduced ($k_{\mathrm{NL}}>0$), AutomataGPT's ability to correctly infer the underlying deterministic rules sharply decreases, indicating that AutomataGPT—trained exclusively on locally governed cellular automata—fails to generalize to systems with nonlocal dependencies. The apparent robustness at $D_{\mathrm{NL}}=1$ occurs possibly because the added nonlocal neighbors coincide with cells already included in the Moore neighborhood, preserving the system's local update structure. Statistical details: 64 samples were evaluated (with reported accuracy averaged over all 64) for each $\left(k_{\mathrm{NL}},D_{\mathrm{NL}}\right)$ pair, with $k_{\mathrm{NL}}\in \left\{1,2,4,8,16\right\},D_{\mathrm{NL}}\in \left\{1,2,4,6,8,10,12,14\right\}$. For cases where $k_{\mathrm{NL}}$ exceeded the number of neighbors at distance $D_{\mathrm{NL}}$, some neighboring cell locations were re‐used in the parity computation.

Since AutomataGPT was trained only on CA with local, deterministic rules, introducing nonlocal state transition dependencies collapsed AutomataGPT's inferred rules matrix application accuracy, averaged over an ensemble of 64 random samples (${\bar{A}}_{\mathrm{IRMA}}$); this is shown in Figure 7, which displays the results of a study in which, for each cell update in accordance with a standard RM, $k_{NL}\in \left\{0,1,2,4,8,16\right\}$ nonlocal neighbors were randomly positioned at a Chebyshev distance $D_{\mathrm{NL}}\in \left\{1,2,4,6,8,10,12,14\right\}$ away and their parity was computed to determine if the deterministic cell update should be flipped. From these pre‐computed orbits, AutomataGPT was tasked with inferring the underlying deterministic rules. The results of this study indicate that $\forall k_{\mathrm{NL}}\neq 0$ AutomataGPT's ${\bar{A}}_{\mathrm{IRMA}}$ sharply decreased relative to baseline ($k_{\mathrm{NL}}=0$), indicating diminished RM inference ability. There was no significant dependence on $D_{\mathrm{NL}}$, aside from the case in which $D_{\mathrm{NL}}=1$, where it was observed that AutomataGPT's performance was improved relative to $D_{\mathrm{NL}}>1,\;\forall k_{\mathrm{NL}}\neq 0$. This intriguing result suggests AutomataGPT demonstrates emergent robustness against minor external ruleset modifications, so long as such modifications to the rule introduce dependencies only within the local (r=1) Moore neighborhood. *This experiment empirically delineates the model's theoretical scope: since AutomataGPT's success depends on the alignment between the orbit‐generation process and the model's assumed local causal structure, it can infer rulesets only when the underlying system itself is locally causal*.

**FIGURE 8:**
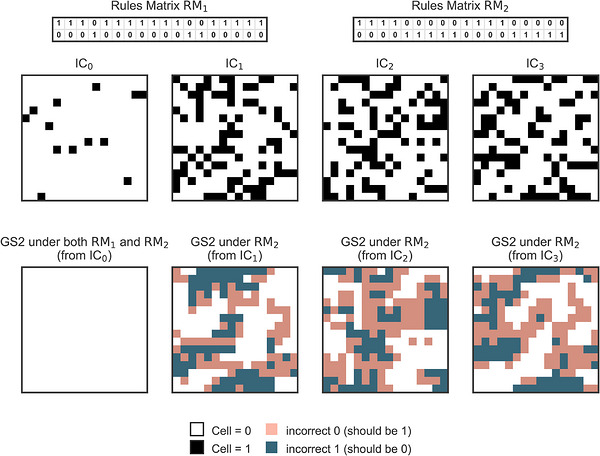
Two distinct RMs (${\mathrm{RM}}_{1}$ and ${\mathrm{RM}}_{2}$) are shown, alongside several ICs (${\mathrm{IC}}_{j},\;j\in \left\{0,1,2,3\right\}$), where both RMs map ${\mathrm{IC}}_{0}$ to the same GS2, but map the other ICs to differing GS2s. Deviations of the ${\mathrm{RM}}_{2}$‐based result and the “correct” ${\mathrm{RM}}_{1}$‐based result are displayed using color. The results indicate that two distinct rulesets may map an IC having sparse metastate coverage to the same GS2, while mapping ICs with high metastate coverage to distinct GS2s. This suggests that IC metastate coverage is a necessary condition to prevent ruleset degeneracy.

Another problem intrinsic to CA systems is that multiple distinct rulesets can both perfectly map some ICs to matching GS2s, while mapping other ICs to distinct GS2s (Figure 8). These degenerate RMs render the inverse problem intractable in the strict sense; finding the “correct” RM is impossible, since there may be multiple equally plausible options. The number of degenerate RMs compatible with a single IC→GS2 transition depends on the number of distinct metastates (neighborhoods) observed in an IC. Specifically, for 2D, binary, deterministic, nondirectional (isotropic) CA, the number of degenerate RMs, $\Omega_{\mathrm{RM}}$, is

(9) $$\Omega_{\mathrm{RM}}=2^{18-N_{M}},$$

where $N_{M}$ is the number of unique metastates in an IC. From Equation (9), it is clear that if $N_{M}=18$, only one RM is possible; likewise, as more metastates go missing from an IC, the number of degenerate RMs increases. We loosely refer to the number of distinct metastates appearing in an IC ($N_{M}$) as “coverage.” In the context long CA orbits, the length of an orbit will generally increase coverage, as each subsequent global state acts a new IC for the one that follows (though long‐orbit coverage increase is not guaranteed to for any IC or RM). Since AutomataGPT only considers a single timestep, we provide an analysis of conditions to ensure coverage in a single IC.

**FIGURE 9:**
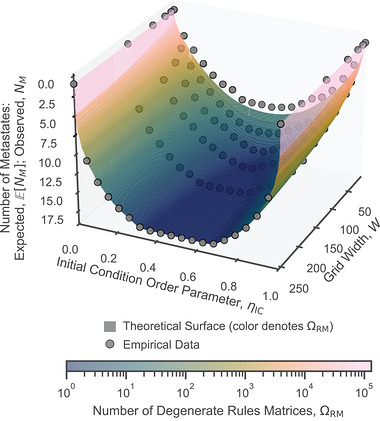
Theoretical surface of the expected number of metastates, $E[N_{M}]$, as a function of initial‐condition order parameter ($\eta_{\mathrm{IC}}$) and grid width (W). Surface color encodes the theoretical degeneracy ${\hat{\Omega }}_{\mathrm{RM}}=2^{\;18-E[N_{M}]}$ on a logarithmic scale. The surface is evaluated on a dense grid with $\eta_{\mathrm{IC}}\in \mathrm{np}.\mathrm{linspace}\left(0,1,500\right)$ and W∈np.linspace(8,256,100) (treated continuously for visualization), using equation 22 and equation 23. Gray points denote empirical means: for each discrete pair $\left(\eta_{\mathrm{IC}},W\right)$ with $\eta_{\mathrm{IC}}\in {\left[0,1\right]}_{20}$ (20 evenly spaced values including endpoints) and W∈{8,16,32,64,128,256}, we find the average the observed number of metastates. Consistent with theory, increased IC disorder and larger grids yield greater metastate coverage and exponentially suppress degeneracy. Statistical details: for experimental data, we generated 30 random binary ICs for each discrete pair $\left(\eta_{\mathrm{IC}},W\right)$ and average the measured number of metastates accordingly.

Figure 9 illustrates this relationship empirically. The theoretical surface shows the expected number of metastates, $E[N_{M}]$, as a function of initial condition order parameter, $\eta_{\mathrm{IC}}$, (the probability of a cell begin in the 1‐state) and grid width, W, with color denoting the corresponding number of degenerate RMs, $\Omega_{\mathrm{RM}}$. Empirical data closely follow our theoretical prediction, confirming that as IC order decreases or grid size increases, coverage increases, and $\Omega_{\mathrm{RM}}$ decreases exponentially. *This degeneracy highlights a fundamental ambiguity in the inverse problem: even assuming perfect RM inference, AutomataGPT can only uniquely identify the generative RM when the IC sufficiently samples the full metastate space, which is more likely for large, disordered ICs*.

Notwithstanding the limitations of our current framework, additional experiments indicate that AutomataGPT can address certain physically relevant inverse problems in a controlled test setting. Specifically, AutomataGPT was able to infer a simple CA ruleset that captures salient short‐horizon features of discretized and binarized Allen–Cahn [88, 89] phase‐separation dynamics (Figure S1). The inferred CA ruleset was shown to reproduce Allen–Cahn behavior on small scales—qualitatively (Figure S2) and quantitatively (Figure S3)– ‐and on large scales (Figure S4) for short time durations. Deviations from ground truth at longer times arise from compounding errors in the recursive CA simulation and from pinning effects caused by the local‐curvature‐insensitive nature of nondirectional radius‐1 rules. These results should therefore be interpreted narrowly: they do not show that arbitrary non‐CA systems admit exact CA descriptions. Rather, they show that, in a controlled setting, a model trained exclusively on synthetic CA trajectories can infer a radius‐1 binary deterministic CA rule that reproduces salient short‐horizon features of a discretized and binarized non‐CA system. A detailed discussion of the methodology, assumptions, results, limitations, and conclusions of this study is provided in the Supporting Information. In particular, Sections S2–S4 detail the Allen–Cahn study itself, while Section S1 places it in a broader conditional framework, clarifying that such non‐CA approximations are meaningful only relative to a chosen coarse‐graining, a fixed surrogate neighborhood class, and the induced local predictive target.

### 2.4 Implications for Future Work

The implications of our work are not limited to the realm of CA research. The AutomataGPT framework shows promise in areas where locality may be reasonably assumed—or where a local representation of an otherwise nonlocal system is desired—and where real‐world data exhibits ample metastate coverage. Thus, AutomataGPT could be the first step toward a new framework for modeling complex systems and systems exhibiting emergence. We show that the state‐transition rules for 2D, binary, deterministic CA can be inferred using AutomataGPT, and that inference accuracy is highly influenced by the breadth of the rules in the training set. Future models trained on CA systems having more states, more spatial dimensions, and larger grids might be able to learn rules across many physical systems, so long as such systems may be effectively coarse‐grained using discrete cells. A key future direction is model selection over surrogate CA rule classes, including neighborhood type and neighborhood radius, since these choices determine both the expressive power of the inferred CA and the degree to which a coarse‐grained non‐CA system can be faithfully approximated. For real data, we envision a simple block‐style renormalization group [90] coarse‐graining: map small patches to single effective cells, preserving locality while compressing token sequences to fit the GPT model context size. The inverse task remains rule discovery, and validation should happen at the same coarse scale where those rules act. While small‐scale information is lost in the coarse‐graining process, larger scale dynamics might become more obvious to the model. Furthermore, long observed sequences can reduce ruleset ambiguity (degeneracy), pointing toward scale‐aware, interpretable rules that capture dominant mechanisms without overfitting to microscopic detail. Accordingly, a pragmatic criterion is whether the inferred coarse rules reproduce the coarse‐grained ground‐truth microdynamics; success would support micro‐to‐macro emergence for a given system, whereas failure would suggest the inferred macro rules are artifacts and that faithful understanding requires the microscopic description. In future work, we will train and evaluate on multi‐step sequences for ruleset extraction to reduce inverse problem ruleset degeneracy through increased metastate coverage and long‐horizon forecasting tasks to enhance forward problem applicability. In addition, we will extend to richer CA (multi‐state, directional/non‐isotropic, stochastic) and coarse‐grained real data'regimes where ruleset identifiability improves with sequence length and direct enumeration becomes impractical.

Such versions of AutomataGPT for forecasting physical systems would have a *three‐fold* advantage over existing AI‐enabled techniques.

1. *Use of Synthetic‐only Training Data*. Since such models would only require examples of time‐evolution for CA systems, datasets could be purely synthetic, eliminating the need for expensive real‐world data collection.
2. *Computational Efficiency*. Due to the high parallelizability of CA algorithms [91], physical simulations represented entirely with CA rules (inferred with future AutomataGPTs) could be efficiently scaled‐up for larger systems.
3. *Simulation Interpretability*. Finding relationships between CA and PDEs could enable a two‐way simulation paradigm in which inferred CA rules could assist researchers with identifying new PDEs, conserved quantities, or other mathematical formalisms to advance understandings for a plethora of physical systems. In fact, Omohundro [92, 93] described a method for converting any 2D nine‐neighbor square‐lattice CA system into a corresponding system of partial differential equations (PDEs). Future work could feasibly synthesize similar methods with AutomataGPT‐based methods to quickly define PDEs for nearly any 2D dynamical system, unlocking a world of dynamical systems analysis across a broad spectrum of scientific disciplines. Alternatively, CA rules could be interpreted intuitively by a human researcher, depending on the particular system at hand, potentially enabling the discovery of new analytic frameworks.

The success of this future research direction will depend on how effectively CA are generally able to simulate complex systems, but we are optimistic considering the wide range of behavior exhibited by CA of differing dimensionalities, state‐possibilities, and rules. Another potential challenge for such a paradigm could be training costs. While GPTs have been shown to scale well with large training sets and context lengths [16, 17, 94], and have architectures, which are highly conducive to training parallelization using GPUs [16], it is not clear exactly how large future versions of AutomataGPT would have to be to capture the complexity present across a plethora of physical systems. Answering such a question will require benchmarking various AutomataGPTs on a range of existing physical systems.

We also note that we did not characterize AutomataGPT's sensitivity to IC distribution type, as we trained all versions of the model using broad‐entropy ICs. It is possible that AutomataGPT carries a latent bias toward and/or against certain groups of ICs; however, we find this possibility unlikely, given the $N_{\text{RM}}=100$ version's unbiased generalization across RMs, since the RMs used for training data also followed a broad‐entropy distribution. Nevertheless, we acknowledge that naturally occurring systems may have different IC and RM distributions than our training data, could reveal previously unknown model biases. Future studies should investigate this possibility, potentially by creating new testing sets comprised of human‐designed CA rulesets and ICs that mimic real systems, or by using real‐world spatiotemporal data in place of CA systems.

While our results demonstrate AutomataGPT's ability to infer rules in a constrained CA setting, its applicability remains tied to the assumption of locality. Many scientific domains, ranging from lattice models in physics and materials science [95, 96] to agent‐based dynamics in biology [97, 98], already rely on locally causal descriptions, so the locality prior aligns with how such systems are commonly understood. Even in other real‐world cases where the balance between local and global influences is unclear, restricting inference to local rules may still be informative: it could highlight dominant mechanisms, provide coarse‐grained approximations, and yield interpretable hypotheses about the system's dynamics. In this sense, AutomataGPT's contribution is not to solve the general inverse problem for arbitrary patterns, but to show how embedding locality into neural architectures enables tractable, interpretable rule discovery in settings where locality is either known or plausibly assumed.

Future work may also benefit from rigorous model interpretation analyses. Deep convolutional models often exhibit an apparent progression from local to global features with depth [99]; it is not clear whether decoder‐only transformers necessarily hard‐wire such hierarchies. Whether coarse patterns emerge in deeper layers (or specific heads) could depend on objectives and supervision. Because our tasks target single‐timestep forecasting and rule inference from short contexts, there is limited pressure to form explicit multi‐scale abstractions. This could change if a future AutomataGPT‐like model were tasked with forecasting and inferring rules for CA at varying levels of coarse‐graining. To probe this, we envision a compact interpretability program that trains sparse autoencoders [100] to surface human‐interpretable units. Such a program could clarify whether the model organizes representations by length‐scale in a CNN‐like manner or instead solves CA tasks without a stable scale hierarchy, informing objectives that encourage interpretable, scale‐aware internal structure.

Another conceptual direction concerns reversibility. In physics, the arrow of time and macroscopic irreversibility emerge despite the reversibility of microscopic laws—this is the famous Loschmidt “paradox” at the heart of the second law of thermodynamics [101]. In CA, however, reversibility has a stricter definition: each configuration must have a unique predecessor. For some classes of CA, such as certain block rules [102] and specific one‐dimensional cases [103, 104], it is possible to prove if a certain ruleset is reversible or not, but in general the problem is undecidable [105]. Accordingly, even if AutomataGPT infers a ruleset, there is no guarantee we can determine whether that ruleset encodes a reversible dynamic. Future studies could examine whether the rules extracted by AutomataGPT tend toward plausibly reversible or time‐symmetric [106] automata by relying on search algorithms to try to determine general trends. Exploring this area could connect AutomataGPT to some of the most fundamental ideas in physics.

### 2.5 Potential Applications

Although our present study focuses on synthetic CA data, the AutomataGPT architecture provides a transferable blueprint for modeling real systems governed by local or quasi‐local interactions. We envision several concrete proof‐of‐concept directions:

1. *Microstructural evolution in materials*. Binary or multi‐state lattice representations of phase transformations, grain growth, or damage evolution could be mapped directly into CA form. AutomataGPT‐inferred rules would then serve as interpretable surrogates for mesoscale simulation, identifying dominant neighbor‐based mechanisms and offering fast parameter sweeps for process design.
2. *Morphogenetic and biological patterning*. Agent‐based or cell‐based datasets can be coarse‐grained into local occupancy grids. AutomataGPT could infer effective developmental rules that describe how local signaling or adhesion translates into global morphology, offering a scalable, data‐driven complement to mechanistic models.
3. *Ecological and reaction–diffusion dynamics*. Spatiotemporal population or concentration maps can be discretized into CA‐like representations, allowing AutomataGPT to recover interpretable rules revealing dominant transport or interaction scales.
4. *Collective robotics and swarm control*. Binary occupancy grids or discretized trajectory fields of robotic swarms can be modeled as CA, where each site encodes agent presence or local orientation. AutomataGPT can be trained on trajectories generated by an existing decentralized control policy, learning an equivalent set of discrete *local* update rules that reproduce the observed collective motion. Recovering a CA representation offers three advantages: (1) it yields a compressed, interpretable abstraction of the swarm's effective policy, (2) it provides a fast surrogate for large‐scale or long‐horizon simulations, and (3) it exposes a transferable local rule grammar that can be modified or redeployed across different swarm embodiments, enabling rapid exploration of new coordination strategies.

These examples illustrate why AutomataGPT remains useful despite its theoretical limits: by focusing on systems where locality is intrinsic or plausible, it transforms a formally intractable inverse problem into a tractable, interpretable, and computationally efficient modeling framework applicable across diverse scientific and engineering domains.

## 3 Conclusion

We demonstrate a novel framework, AutomataGPT, for forecasting time‐evolution (forward problem) and inferring rules (inverse problem) for 2D binary deterministic r=1 CA systems with toroidal boundary conditions. We observe that increasing the number of distinct rules used to create the training set significantly improves model performance on both the forward and inverse problems. Our findings suggest a promising and interpretable direction for artificial intelligence‐assisted symbolic modeling under explicit structural priors. AutomataGPT, trained exclusively on *synthetic‐only* data, accurately forecasts and infers rules from previously unseen 2D CA systems. Additionally, AutomataGPT shows stark improvements in performance and reductions in inductive bias for systems outside of its training set after being trained on increasingly wide sections of rule space. This framework may be useful in future applications to infer effective local rules for certain real‐world systems whose coarse‐grained dynamics are reasonably approximable within a chosen CA rule class, as supported here by a controlled proof‐of‐concept application to discretized phase‐separation dynamics governed by the Allen–Cahn equation. Future work is required to rigorously characterize the dependence of such inference on surrogate rule‐class selection, including neighborhood type and radius, and on which classes of data and dynamical systems are most amenable to this form of symbolic modeling. Such a paradigm might dramatically reduce the costs (both monetary and temporal) of training AI models for physical prediction tasks. We believe it is crucial that future work be done to investigate the scaling behavior of AutomataGPT‐like models, so as to determine the feasibility of training such models using larger CA rule spaces with more degrees of freedom (larger grid sizes, more cell states, and higher dimensionalities). If there exists enough latent complexity within large rule spaces, then future models might be able to learn to extract meaningful rules from a wide range of systems across varying length scales, so long as empirical time‐evolution data can be provided using a prompt. The implications of AutomataGPT extend beyond CA, hinting at broader, transdisciplinary applications for which GPT‐based architectures could be used not only to infer the behavior of systems, but to extract meaning in a way that advances human understanding, paving the path for human‐AI symbiosis for scientific advancement. In that vein, AutomataGPT exemplifies a promising direction in bridging symbolic rule‐based systems and subsymbolic neural architectures. *The key concept put forth here is that by enabling interpretable rule inference alongside flexible forecasting, it paves the way for hybrid models that combine the transparency of cellular automata with the expressive power of large language models*. Our findings support the idea that AI models need not merely fit or predict empirical data; they can also recover underlying generative mechanisms, akin to discovering governing equations. This suggests a path toward AI‐native scientific frameworks in which models do not just simulate, but understand and re‐express the rules of complex systems.

## 4 Experimental Section

### 4.1 Model Architecture and Training Hardware

AutomataGPT was constructed in Python using x‐transformers [107]. The models in this study were trained on a workstation equipped with a high‐end CUDA‐compatible GPU (RTX A4000, NVidia, Santa Clara, CA, USA).

### 4.2 Hyperparameters used for training

Hyperparameters were initially selected heuristically for optimal performance, as the GPU primarily used for training (RTX A4000, NVidia, Santa Clara, CA, USA) had 16 GB of VRAM. Unless otherwise stated, all instances of AutomataGPT used the following set of hyperparameters during training, as described in Table 1.

**TABLE 1:** AutomataGPT's hyperparameters.

*Hyperparameter*	*Value*
num_tokens	22
max_seq_len	555
dim ($d_{\mathrm{model}}$)	256
depth ($N_{\mathrm{layers}}$)	6
heads (h)	4
RPE	True
Flash attention	True
Optimizer	Adam
Learning rate	1e‐4
mask_prob	0.15
Loss function	Cross entropy loss (CLE)
Batch size	50
Gradient accumulation period	1
Training data IC ordering	broad‐entropy
Warm‐up scheduler	linear

### 4.3 Model versions

A full list of all AutomataGPT model version used in this work is provided in Table 2.

**TABLE 2:** Summary of all AutomataGPT versions featured in this study, detailing their key characteristics and problem type.

*Problem type*	$N_{\text{RM}}$	*Model name*	*Grid size*	*Grid topology*	*Deterministic CA?*	*Neighborhood*	*# Cell states*
Forward	2	AutomataGPT‐2DBD‐2rf	16×16	2D Toroidal	Yes	Moore (r=1)	2
Forward	10	AutomataGPT‐2DBD‐10rf	16×16	2D Toroidal	Yes	Moore (r=1)	2
Forward	100	AutomataGPT‐2DBD‐100rf	16×16	2D Toroidal	Yes	Moore (r=1)	2
Inverse	2	AutomataGPT‐2DBD‐2ri	16×16	2D Toroidal	Yes	Moore (r=1)	2
Inverse	10	AutomataGPT‐2DBD‐10ri	16×16	2D Toroidal	Yes	Moore (r=1)	2
Inverse	100	AutomataGPT‐2DBD‐100ri	16×16	2D Toroidal	Yes	Moore (r=1)	2

### 4.4 Training loop

AutomataGPT‐2DBD‐2rf, ‐10rf, ‐2ri, and ‐10ri models were trained for 50 epochs, and AutomataGPT‐2DBD‐100rf and ‐100ri were trained for 20 epochs. For each batch within each epoch, training loss was calculated, followed by subsequent backpropagation. Then validation loss was calculated without calculating gradients, since this loss was not used for weight adjustment. After each epoch of training, AutomataGPT was tasked with inference—which either required predicting the GS2 or the RM, depending on the model version/problem type—and accuracies were calculated across all samples in the testing set. For each batch, the corresponding epoch, global step, training loss, validation loss, testing accuracy, and elapsed time were logged.

### 4.5 Data Generation Overview

To generate training sets, validation sets, and testing sets, a custom Python script was used. First binary, deterministic RMs were stochastically generated. Then, IC game states were generated stochastically as a 2D, 16 × 16 numpy [108] arrays. Then, the corresponding GS2 for every previously generated IC, based on each corresponding RM, was calculated. Lastly, each IC‐GS2‐RM triad was stored in a numpy array. When training or testing models, these numpy arrays were reformatted into lists of strings, with the relative ordering of the ICs, RMs, and GS2s depending on if the dataset was intended for the forward problem or inverse problem (Figure 2). For the purpose of model performance characterization across all versions of AutomataGPT, a single testing set was used with dataset parameters $N_{\text{RM}}=100;N_{\text{IC}}=2$.

### 4.6 Data Topology

Each untokenized sample in the training data (or validation/testing data) was represented as a 1D string where the rules matrix, IC‐matrix, and GS2‐matrix were flattened and concatenated in varying orders, depending on the training task.

### 4.7 RM Formalization

**FIGURE 10:**
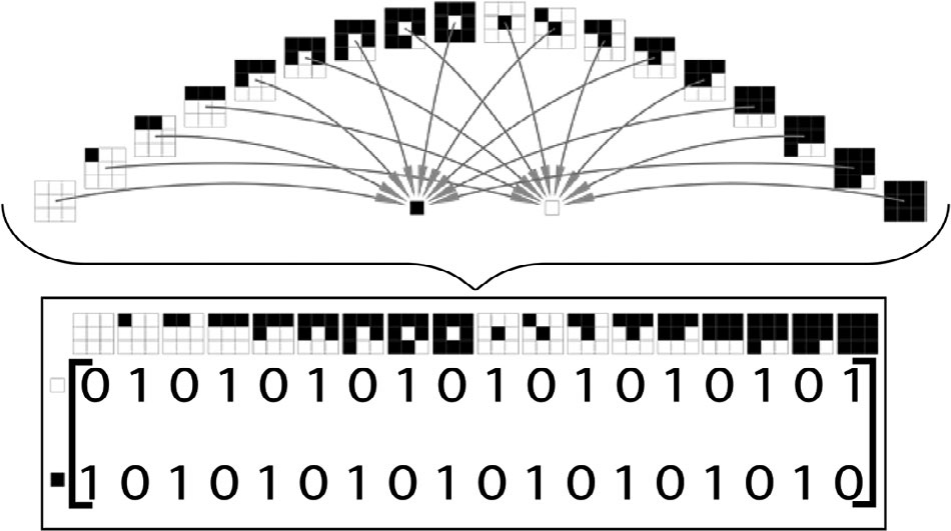
Transformation of a graph representation of state transition in 2D binary CA to a 2D binary adjacency matrix. We refer to the latter mathematical object as a “rules matrix”.

As shown in Figure 10, an unweighted directional graph representing state transition for binary, deterministic CA may be losslessly represented as a binary adjacency matrix [109]. The rows of this matrix correspond to the final state of the cell undergoing transition, and the columns represent the possible initial “metastates” of the cell. We define a metastate as a 3×3 binary matrix representing the state of a cell along with its local (r=1) Moore neighborhood [58]. In other words, the metastate is a combination of the state of a cell and all of its neighbors. We assume metastates are permutation invariant, meaning the orientation of neighboring states does not change the identity of the metastate so long as the net number of neighboring cells in each possible state remains unchanged. Since binary (r=1) 2D automata have 18 metastates and 2 individual cell states, we defined RMs as 2×18 2D arrays.

### 4.8 Calculating the Total Number of Possible RMs

In a 2D deterministic binary (isotropic/count‐based) CA with a Moore neighborhood, each cell can be in one of two possible states: 0 or 1. The next state of the cell depends on its current state and the number of neighbors in state 1. Because there are 8 neighbors, the neighbor count can range from 0 to 8, giving us

(10) Possible neighbor counts=9.

Also considering the cell's current state (two possibilities: 0 or 1) brings the total to

(11) Total metastates=2×9=18.

A *deterministic* rules matrix (RM) specifies the next state (either 0 or 1) for *each* of these 18 configurations. Hence, for every metastate there are 2 possible next states, and thus the total number of possible deterministic RMs is $2^{18}$. Therefore, for a 2D binary CA (r=1 Moore neighborhood and non‐directional rules), there are exactly $2^{18}$ distinct RMs.

100 RMs, for instance, represents only a tiny fraction of the entire rule space:

(12) $$\begin{array}{cc} & \text{Percentage of total rule space for 100 RMs}\\ & \;=100\%\times \frac{100}{2^{18}}≅0.03814\%\approx 0.04\%. \end{array}$$

### 4.9 RM Generation

For the present work, we generate rule matrices (RMs) in a *deterministic* manner by choosing exactly one next state for each *metastate*. While our Python script also offers a stochastic mode, it was not used in this study. In addition, each RM is labeled with an integer *Rules Matrix ID* (RMID), yet in the current implementation the ID is *not* used to select the next states. Instead, these are chosen randomly at runtime.

### 4.10 Deterministic Rule Assignment

For each column (metastate) j∈{1,⋯,c}, we *randomly* choose a next state (in {0,⋯,b−1}) via random.randint(0, b‐1). Formally, for each column j,

(13) $$\text{RM}\left[k,j\right]\;=\;\left\{\begin{array}{cc} 1 & \text{if}\;k=\mathrm{rand}\_{\mathrm{state}}_{j},\\ 0 & \text{otherwise}, \end{array}\right.$$

where $\mathrm{rand}\_{\mathrm{state}}_{j}$ is a random integer from [0,⋯,b−1]. As a result, each metastate has *exactly one* valid next state, but the choice of which one is random. Although this is still a fully deterministic rule (each metastate maps to a single next state), it is drawn stochastically at the time of generation.

### 4.11 Stochastic Mode (not used)

Our code also supports a purely *stochastic* mode, in which each column can encode a probability distribution over the possible next states. However, all results in this paper exclusively use the deterministic mode described above. (We anticipate that the stochastic mode will be useful for future studies on stochastic CA.)

### 4.12 IC Generation

The initial states of each cellular automaton are generated through a stochastic process involving two main steps: random order parameter generation and cell‐by‐cell state assignment.

First, for each cellular automaton, we generate a set of random order parameters that define a probability distribution over the possible cell states. Specifically, we generate b−1 random numbers (where b is the base of the automaton), sort them in ascending order, and extend this list by including 0 at the beginning and 1 at the end. This results in a sequence:

(14) $$p_{0}=0<p_{1}<p_{2}<\cdots <p_{n-1}<p_{n}=1.$$

The order parameters are defined as the absolute differences between consecutive elements

(15) $$\Delta p_{k}=p_{k+1}-p_{k},\;\text{for}\;k=0\;\text{to}\;n-1.$$

$\Delta p_{k}$ represents the probabilities of assigning state k to a cell, such that $\sum_{k=0}^{n-1}\Delta p_{k}=1$.

Second, each cell in the cellular automaton grid is assigned a state based on these probabilities. For each cell, we generate a random number η uniformly from [0,1]. We then determine the state k of the cell by finding the smallest k such that

(16) $$\eta <\sum_{i=0}^{k}\Delta p_{i}.$$

### 4.13 GS2 Generation

GS2s were generated by applying a deterministic rules matrix (RM) to each cell in an initial configuration (IC). The total number of *metastates* was denoted by c. For every cell $S_{\left(i,j\right)}$, the following procedure was carried out:

1. *Metastate Identification*. The metastate $\mu_{\left(i,j\right)}\in \left\{1,\cdots ,c\right\}$ was determined by combining the current state of $S_{\left(i,j\right)}$ with the number of its neighbors in state 1. This operation was count‐based (isotropic), and $\mu_{\left(i,j\right)}$ was defined as
(17) $$\begin{array}{cc} \mu_{\left(i,j\right)}\; & =\;1\;+\;\underset{\begin{array}{c} \text{shifts index by}\;9\\ \text{if cell state}=1 \end{array}}{\underset{︸}{\left(\text{current state of}\;S_{\left(i,j\right)}\right)\cdot 9}}\;\\ & \;+\;\underset{\in \{0,\cdots ,8\}}{\underset{︸}{\left(\#\text{of neighbors in state}\;1\right)}}. \end{array}$$
  Thus, $\mu_{\left(i,j\right)}$ took values in {1,⋯,9} if $S_{\left(i,j\right)}$ was in state 0, or in {10,⋯,18} if $S_{\left(i,j\right)}$ was in state 1.
2. *Rules‐Matrix Application*. Each column of the RM, $\text{RM}[\cdot ,\mu_{\left(i,j\right)}]$, encoded a deterministic mapping from $\mu_{\left(i,j\right)}$ to a single next state in {0,1}. Because the RM was binary and deterministic, exactly one of its two rows was set to 1 for each column, and the other was 0. Concretely,
(18) $$\text{next state of}\;S_{\left(i,j\right)}\;=\;\left\{\begin{array}{cc} 0 & \text{if RM}[0,\mu_{\left(i,j\right)}]=1,\\ 1 & \text{if RM}[1,\mu_{\left(i,j\right)}]=1. \end{array}\right.$$
3. *Formation of the GS2 Configuration*. Once the next state of every $S_{\left(i,j\right)}$ was computed, these new states were assembled into a two‐dimensional array of the same dimensions as the IC. This array was referred to as GS2.

Hence, GS2 was generated by mapping each cell's current state and neighborhood (its metastate) to a single next state through the RM. This process was repeated for all cells, yielding the complete evolved configuration after one timestep.

### 4.14 Instruction Tuning

To incorporate the time‐progression of each CA system into our training set, we represented the *RM*, the *IC*, and the resulting *GS2* as a *single string* with special tokens marking their boundaries. Concretely, after simulating the CA for one time‐step (or more, depending on the experiment), we flattened each 2D matrix into a one‐dimensional list and converted it to a space‐delimited string. We then concatenated the three core elements (RM, IC, GS2) using reserved tokens as delimiters. An example format for the forward problem appears below:

[BOS] [R] RM [BIC] IC [EIC] [BGS2] GS2 [EGS2] [EOS],

where:

- [BOS] and [EOS] mark the start and end of the entire sequence.
- [R] introduces the flattened *RM*.
- [BIC] (begin IC) and [EIC] (end IC) delimit the flattened *IC*.
- [BGS2] (begin GS2) and [EGS2] (end GS2) delimit the *GS2*.

This preprocessed string was then tokenized to form the input vector used for training.

### 4.15 Data Splitting

After a dataset of samples formatted for the relevant inference tasks of the corresponding model was stochastically generated, this dataset was shuffled using the .shuffle() function in the random Python module. After shuffling, the initial 90% of samples were assigned to the training dataset, the next 9% to the validation dataset, and the final 1% to the testing set.

### 4.16 Tokenization

We employed a custom tokenizer based on the tokenizers Python module [110] that operates on strings of specific characters and groups of characters, separated by spaces. By predefining the vocabulary to only include necessary symbols for our specific type of data, we could drastically reduce the vocabulary size to only 22 unique tokens. We suspect that this small vocabulary size improved training efficiency compared to our previous LifeGPT model [35] which used UTF‐8‐based tokenization. The tokenization process produced token indices that were subsequently transformed into vector representations via an embedding operation in our models.

### 4.17 Forward Problem Inference

Inferences were performed in both forward and inverse modes to evaluate and validate model predictions under minimal prior assumptions. The procedure consisted of generating outputs from a pretrained model and comparing these outputs to ground truth data, either in the form of binary strings or rule matrices. The core methods are outlined below.

Forward inference was performed by iterating over a dataset of samples and extracting relevant segments from each sample. The model autoregressively generated an output string representing GS2 for every input. The following steps were used to compute a single accuracy metric per sample:

1. *Prompting and Inference*. Tokens corresponding to the ICs and RMs of testing set samples were extracted, tokenized, and provided to AutomataGPT. AutomataGPT then generated (with temperature set to 0) an inferred GS2, which was subsequently decoded into a human‐readable string.
2. *Binary Extraction*. The decoded output was filtered to isolate only the binary data (0 and 1), and the ground truth underwent the same filtering process.
3. *Accuracy Computation*. A custom script was employed to compare predicted and ground‐truth binary data, yielding an accuracy value for each sample.
4. *Accuracy Logging*. The per‐sample accuracies were appended to a log file, and a running average was maintained throughout the process. Finally, after the last sample was evaluated, an average forward accuracy was computed across all samples.

### 4.18 Inverse Problem Inference

Inverse inference was the process of inferring an RM from an IC and GS2 pair, and validating the RM's accuracy by both comparing it with the ground truth RM and applying it to the sample IC to assess the accuracy of the resulting computed GS2. Each sample yielded two distinct accuracy metrics: one for the RM itself ($A_{\text{RMI}}$) and another for the application of the inferred RM to compute GS2 ($A_{\text{IRMA}}$). The procedure was as follows:

1. *Prompting and Inference*. Tokens corresponding to the ICs and GS2s of testing set samples were extracted, tokenized, and provided to AutomataGPT. AutomataGPT (with temperature set to 0) generated an inferred RM, which was decoded and reshaped into a (2×18) array.
2. *RM Validation*. The predicted RM was checked to ensure that each column's entries summed to 1, thereby confirming that valid transition probabilities were encoded. If the inferred RM was found to be illogical in this regard, both accuracy metrics ($A_{\text{RMI}}$ and $A_{\text{IRMA}}$) were set to 0 for the corresponding sample.
3. *Evolution to GS2*. If validation succeeded, the IC was updated for one timestep using the inferred RM. The resulting configuration (computed GS2) was compared against the ground truth GS2 to compute the inferred RM application accuracy ($A_{\text{IRMA}}$). $A_{\text{RMI}}$ was set to 1 if $A_{\text{IRMA}}$ was also found to be 1 (indicating a degenerate solution), and otherwise it was set to the ratio of correctly inferred RM tokens to total RM length (see Equation 3).
4. *Accuracy Logging*. Both $A_{\text{RMI}}$ and $A_{\text{IRMA}}$ were recorded for each sample in a log file. Average inverse problem accuracies were then computed (see Equations 5 and 6).

### 4.19 Orbit Divergence Testing

Orbit divergence testing involved inferring RMs from IC, GS2 pairs in a testing set (50 unique CA orbits, each using a stochastically generated RM), comparing with the ground truth RMs to compute the strict rules matrix inference accuracies ($A_{\text{RMI, strict}}$), and subsequently computing orbits from each IC using the corresponding inferred RM. Then, those computed orbits were compared to the ground truth orbits (computed from ICs using the ground truth RMs found in the testing set) to find the in‐orbit rules matrix application accuracy for each game state g, $A_{\text{IRMA}}\left(g\right)$.

### 4.20 Locality‐Break Testing

This experiment probed AutomataGPT's sensitivity to departures from strictly local, deterministic dynamics. All simulations were performed using Exp1_LocalityBreak.ipynb, which imports shared utilities from _shared/ca_utils.py for rule generation, orbit computation, and stochastic IC sampling. Standard 2D binary cellular automata were evolved under a Conway‐like local rule (life_like_rule([3],[2,3])), and nonlocal perturbations were introduced by flipping each cell's deterministic update based on the parity of a set of $k_{\mathrm{NL}}$ randomly selected remote neighbors positioned at a Chebyshev distance $D_{\mathrm{NL}}$. The resulting system remains deterministic but gains long‐range dependencies. For each $\left(k_{\mathrm{NL}},D_{\mathrm{NL}}\right)$ configuration, 64 random initial conditions (ICs) of size 16×16 were generated and evolved one step to yield paired IC→GS2 transitions, from which AutomataGPT inferred candidate rule matrices.

Inference performance was quantified using the mean inferred‐rules‐matrix application accuracy, ${\bar{A}}_{\mathrm{IRMA}}$, across all samples. As shown in Figure 7, ${\bar{A}}_{\mathrm{IRMA}}$ sharply decreased for all $k_{\mathrm{NL}}\neq 0$, indicating a strong dependence on local causal structure. For that figure, surface smoothing was achieved with UniformTriRefiner, setting subdiv = 3 for smooth shading and constraining the $k_{\mathrm{NL}}=0$ ridge to the baseline; no additional pos‐thoc filtering was performed.

### 4.21 Demonstrating Non‐Uniqueness of Rulesets

The second experiment investigated the intrinsic non‐uniqueness of the CA inverse problem, where multiple distinct rules matrices (RMs) can reproduce identical state transitions for certain ICs. Implemented in Exp2_3_NonUniqueness_and_Rule_Cardinality.ipynb, this setup builds a reference RM (${\mathrm{RM}}_{1}$) using life_like_rule([3],[2,3]) and a degenerate variant (${\mathrm{RM}}_{2}$) via flip_unobserved_columns(), which inverts all unobserved metastates from ${\mathrm{RM}}_{1}$ according to a mask computed by observed_metastate_mask(). The sparse reference ${\text{IC}}_{0}$ was chosen such that only a subset of the 18 possible local neighborhoods appeared, leaving ${\mathrm{RM}}_{1}$ and ${\mathrm{RM}}_{2}$ indistinguishable when applied to ${\text{IC}}_{0}$. To demonstrate divergence, denser ICs (${\mathrm{IC}}_{B,i}$) were generated with higher densities until their GS2 states differed under ${\mathrm{RM}}_{1}$ and ${\mathrm{RM}}_{2}$. Each experiment consisted of (i) evolving ${\text{IC}}_{0}$ and ${\mathrm{IC}}_{B,i}$ once under both RMs, (ii) recording GS2 states, and (iii) computing per‐cell mismatches between the two outcomes.

### 4.22 Quantifying Degenerate RM Cardinality

The third experiment quantified how metastate coverage constrains the uniqueness of rule inference. For 2D binary isotropic CA with radius r=1, there exist 18 possible local metastates, each corresponding to a column in the rule matrix. For a given IC with $N_{M}$ unique metastates, the number of degenerate rules consistent with that IC is given by

(19) $$\Omega_{\mathrm{RM}}=2^{18-N_{M}}.$$

Here, coverage refers to the fraction of unique metastates observed in the IC. AutomataGPT performs one‐step inference, so sufficient metastate diversity within a single IC is critical for reducing degeneracy.

We derived a theoretical relationship between $E[N_{M}]$, the IC order parameter $\eta_{\mathrm{IC}}$ (the probability that a cell is in the 1‐state), and grid width W. For each $\left(\eta_{\mathrm{IC}},W\right)$ pair, random ICs were generated using generate_random_ic(), metastate masks were computed using observed_metastate_mask(), and $\Omega_{\mathrm{RM}}$ was obtained via degeneracy_cardinality_from_mask(). Empirical averages were compared to an analytical prediction, $E\left[N_{M}\right]\left(\eta_{\mathrm{IC}};W^{2}\right)$, obtained by treating neighborhoods as independent Bernoulli draws.

We make the isotropic (r=1 Moore) assumption that a local neighborhood is fully specified by the pair (c,k), where c∈{0,1} is the central‐cell state and k∈{0,⋯,8} is the number of 1s among the eight neighbors. This yields |M|=2×9=18 possible metastates. Under a Bernoulli IC with order parameter $\eta_{\mathrm{IC}}=P\left\{\;\text{cell}=1\;\right\}$, neighborhoods at different lattice sites are modeled as independent and identically distributed draws from the categorical distribution over the set of all metastates, M. Letting, N be the number of neighborhoods sampled in an W×W grid, we take $N\approx W^{2}$ to be true since our grid is toroidal.

For a fixed metastate (c,k), the single‐draw probability is the product of the center‐state probability and the binomial count for the neighbors:

(20) qc,k=P(c,k)=ηICc,1−ηIC1−c8kηICk,1−ηIC8−k.

Let $X_{c,k}$ be the indicator that metastate (c,k) appears at least once among the N draws. Under the independence approximation across sites,

(21) $$P\left\{X_{c,k}=1\right\}\;=\;1-{\left(1-q_{c,k}\right)}^{N}.$$

The number of distinct metastates observed in the IC is $N_{M}=\sum_{(c,k)\in M}X_{c,k}$. Taking expectations and using linearity yields the plug‐in estimator used in our study:

(22) E[NM](ηIC,W)=∑c∈{0,1}∑k=081−(1−qc,k(ηIC))W2,qc,k(ηIC)=ηICc(1−ηIC)1−c8kηICk(1−ηIC)8−k.

Finally, plugging this expectation into the degeneracy relation for 2D, isotropic, binary, r=1 CA ( |M|=18 ) yields the analytical predictor

(23) $${\hat{\Omega }}_{\mathrm{RM}}\left(\eta_{\mathrm{IC}};W\right)\;=\;2^{\;18-E\left[N_{M}\right]\left(\eta_{\mathrm{IC}};W\right)}.$$

### 4.23 Generative AI Use

Some Python scripts used for data generation, model training, data processing, and figure generation were written with the assistance of GPT‐3.5, GPT‐4, and GPT‐4o from OpenAI. All scripts generated/edited in this manner were carefully reviewed, validated, and manually corrected, in the event of errors, by an author prior to implementation in our work.

### 4.24 Codes and Data Availability

All codes and data are available for non‐commercial use at: https://github.com/lamm‐mit/AutomataGPT.

### 4.25 Statistical Analysis and Plotting

All analyses were performed in Python (v. 3.12.3) within a conda environment. Core packages and their versions were: NumPy (v. 1.26.4), pandas (v. 2.2.2), SciPy (v. 1.14.1), seaborn (v. 0.13.2), and Matplotlib (v. 3.9.2). Colormaps were generated using cmap (v. 0.4.0). Machine learning models were implemented with PyTorch (v. 2.5.0, CUDA 12.4, cuDNN 9.0), torchvision (v. 0.20.0), and torchaudio (v. 2.5.0) using x‐transformers (v. 1.40.3).

No data transformation or normalization was applied, and no outliers were excluded. Continuous variables (model accuracy values) are reported as mean values; fractions of perfect accuracy ($f_{p}$) and error ($f_{e}$) were also calculated. The sample size for each model configuration was n=200 test cases.

Accuracy distributions were summarized into ten bins [0.0,0.1),⋯,[0.9,1.0], and bar plots report the relative frequencies of samples per bin. Associations between rules‐matrix distances ($d_{H}$, $d_{J}$, JSD) and accuracy were assessed using ordinary least‐squares linear regression (two‐sided). Reported values include slope, intercept, and coefficient of determination ($R^{2}$). To avoid ceiling/floor effects, observations with boundary accuracies (A=0 or 1) were excluded a priori from the linear regressions used to report $R^{2}$ in the inductive‐bias testing figures (Forward and Inverse); all points remain visible in the scatter plots for context.

All statistical analysis and figure generation were performed in Python. Statistical significance thresholds (p‐values) were not applied, as the analysis is descriptive.

## Conflicts of Interest

The authors declare no conflict of interest.

## Supporting information


**Supporting File 1**: advs75040‐sup‐0001‐SuppMat.pdf.

**Supporting File 2**: advs75040‐sup‐0002‐Data.zip.

## Data Availability

The data that support the findings of this study are available in the supplementary material of this article.
