# Supplementary material for: AutomataGPT: Transformer‐Based Forecasting and Ruleset Inference for Two‐Dimensional Cellular Automata
Source: Adv Sci (Weinh). 2026 Apr 9;13(33):e11352. doi: 10.1002/advs.202511352 (PMC13271642; doi:10.1002/advs.202511352)
Supplement: Supplementary file 1 — Supporting File 1: advs75040‐sup‐0001‐SuppMat.pdf. [file ADVS-13-e11352-s002.pdf]

Supporting Information

AutomataGPT: Transformer-Based Forecasting and Ruleset Inference for Two-Dimensional Cellular Automata

Jaime A. Berkovich, Noah S. David, and Markus J. Buehler*

S1 Theoretical Framework for Pairwise Local-Rule Inference from Orbits

In this section, we formalize the inverse problem studied in this work: inferring a *local* cellular-automaton (CA) ruleset, equivalently represented as a rules-matrix (RM), from spatiotemporal data when the inverse model is queried using *pairwise* samples of a trajectory—namely, initial-condition to game state 2 (IC–GS2) pairs, as in our protocol. The purpose of the derivations is to distinguish between two separate modeling choices: first, the coarse-graining and discretization that induce a discrete-state lattice process; and second, the surrogate CA neighborhood radius r that defines the local hypothesis class used for inference. Within that framework, we then clarify how IC–GS2 sampling and neighborhood coverage control identifiability in the deterministic-CA setting, and how, more generally, a coarse-grained non-CA system may admit an *effective local surrogate* within a chosen CA rule class.

All theoretical statements below are *conditional* on a chosen coarse-graining/discretization procedure and a fixed local rule class. Accordingly, this section does *not* address joint discovery of the correct rule family, neighborhood type, or radius from data; rather, it clarifies what can and cannot be inferred *within* a specified local hypothesis class.

S1.1 Discrete-State Representation via Coarse-Graining and Discretization

Let $\{U^t\}_{t\geq 0}$ denote an underlying trajectory, which may be a continuous field or a higher-state discrete field. We choose a coarse-graining map G (spatial and/or temporal) together with a discretization map Q (e.g., thresholding or binning), and define the induced discrete-state lattice trajectory by

$$\mathbf{x}^t := Q\big(G(U^t)\big) \in \{0, 1, \ldots, n-1\}^{\Lambda}, \tag{S1}$$

where $\Lambda = \{1, \ldots, N\}^2$ denotes the lattice and $n \geq 2$ is the number of discrete states. The inverse problem considered throughout this section is then: given samples from $\{\mathbf{x}^t\}$, infer a local update rule that predicts $\mathbf{x}^{t+1}$ from $\mathbf{x}^t$.

This representation should be interpreted as a *modeling choice*. Different choices of G and Q induce different discrete processes and therefore different inverse problems. All subsequent identifiability and approximation statements are relative to the particular induced lattice process in Equation (S1).

S1.2 Neighborhoods, Local Rules, and the Forward Update Operator

We fix a neighborhood shape $\mathcal{N}_r \subset \mathbb{Z}^2$ of radius r and define its size as $m := |\mathcal{N}_r|$. For any lattice state $\mathbf{x} \in \{0, \ldots, n-1\}^{\Lambda}$ and any site $u \in \Lambda$, we define the local neighborhood configuration by

$$\eta_r(\mathbf{x}, u) \in \mathcal{S} := \{0, 1, \ldots, n-1\}^m. \tag{S2}$$

Thus, $\mathcal{S}$ is the set of all neighborhood types available under the chosen radius and neighborhood geometry. We define a deterministic local ruleset as a function

$$f : \mathcal{S} \to \{0, 1, \ldots, n-1\}, \tag{S3}$$

and the induced synchronous CA forward operator $\mathcal{C}_f$ by the componentwise update

$$\left(\mathcal{C}_f(\mathbf{x})\right)_u := f(\eta_r(\mathbf{x}, u)), \qquad \forall u \in \Lambda. \tag{S4}$$

All statements below are conditioned on this fixed pair $(r, \mathcal{N}_r)$ and the associated hypothesis class of local rules (Equation (S3)). In particular, identifiability from data is always meant *within this chosen local rule class*. Changing r changes the neighborhood space $\mathcal{S}$ and therefore changes both the inverse problem and the best achievable approximation error.

S1.3 IC–GS2 Pairwise Sampling from Orbits

We now formalize the pairwise sampling protocol used throughout this work. Let $N_p \geq 1$ be the number of sampled pairs. For each $p \in \{1, \ldots, N_p\}$, we sample an orbit time index t_p and define the IC–GS2 pair by

$$\mathbf{X}^{(p)} := \mathbf{x}^{t_p}, \qquad \mathbf{Y}^{(p)} := \mathbf{x}^{t_p+1}. \tag{S5}$$

From this pair, we define the multiset of sitewise transitions

$$\mathcal{D}^{(p)} := \left\{ \left(\eta_r(\mathbf{X}^{(p)}, u),\ \mathbf{Y}^{(p)}_u \right) : u \in \Lambda \right\}, \tag{S6}$$

which makes explicit the local constraints that the pair imposes on any candidate rule.
We also define the set of neighborhood types observed in the IC of pair p by

$$\mathcal{S}^{(p)}_{\text{obs}} := \left\{ \eta_r(\mathbf{X}^{(p)}, u) : u \in \Lambda \right\} \subseteq \mathcal{S}, \tag{S7}$$

and define the corresponding missing set and missing count as

$$M^{(p)} := \mathcal{S} \setminus \mathcal{S}^{(p)}_{\text{obs}}, \qquad q^{(p)} := |M^{(p)}|. \tag{S8}$$

The quantity $q^{(p)}$ measures how underdetermined a deterministic inverse problem remains when only the single pair $(\mathbf{X}^{(p)}, \mathbf{Y}^{(p)})$ is available. Intuitively, every neighborhood type not observed in the IC leaves one portion of the local rule table unconstrained.

S1.4 Per-Pair Forward Accuracy

Given any deterministic rule f, we define its empirical forward accuracy on pair p by

$$\widehat{A}_p(f) := \frac{1}{|\Lambda|} \sum_{u \in \Lambda} \mathbf{1} \left\{ \left(\mathcal{C}_f(\mathbf{X}^{(p)}) \right)_u = \mathbf{Y}^{(p)}_u \right\} \in [0, 1]. \tag{S9}$$

This quantity measures one-step agreement between the predicted next state $\mathcal{C}_f(\mathbf{X}^{(p)})$ and the ground-truth GS2 $\mathbf{Y}^{(p)}$ for the specific source pair p.

S1.5 Pairwise Inference and Sample-Best Selection

Let Infer$(\cdot,\cdot)$ denote a learned inverse operator that maps an IC–GS2 pair to a deterministic CA rule (equivalently, to an RM encoding that rule). For each pair p, we define the inferred rule

$$\hat{f}^{(p)} := \text{Infer}\Big(\mathbf{X}^{(p)}, \mathbf{Y}^{(p)}\Big) . \tag{S10}$$

We then define the sample-best inferred rule by self-scoring:

$$\hat{f}_{\text{best}} := \arg \max_{1 \le p \le N_p} \widehat{A}_p\Big(\hat{f}^{(p)}\Big) . \tag{S11}$$

This selection rule is a practical "best-of-N_p" heuristic: each candidate rule is produced from one source pair and then evaluated on that same pair. Accordingly, the score $\widehat{A}_p(\hat{f}^{(p)})$ should be interpreted as a measure of *within-pair consistency*, not as an unbiased estimate of out-of-sample or long-horizon generalization. The importance of this selection procedure is operational: when multiple pairwise candidates are available, self-scoring increases the chance of retaining at least one high-quality candidate, as formalized later on in Section S1.11.

S1.6 Deterministic CA Inverse Learnability via Pairwise Coverage

We first analyze the exact deterministic-CA setting. The goal here is to isolate the intrinsic ambiguity of inferring a *deterministic* local rule from a single IC–GS2 pair. The central observation is that a pair can constrain the rule only on neighborhood types that actually appear in the IC, namely $\mathcal{S}^{(p)}_{\text{obs}}$ in Equation (S7).

Assumption (Deterministic CA generator). Assume there exists a "true" deterministic rule $f^\star : \mathcal{S} \to \{0, \dots, n-1\}$ such that the trajectory satisfies

$$\mathbf{x}^{t+1} = \mathcal{C}_{f^\star}(\mathbf{x}^t) \quad \forall t. \tag{S12}$$

Under this assumption, every observed neighborhood type has a well-defined deterministic next-state assignment, so pairwise consistency is well-defined.

Definition (Pairwise consistency set). For pair p, define the set of deterministic rules consistent with the observed sitewise transitions by

$$\mathcal{F}^{(p)} := \Big\{ f :\ f(s) = f^\star(s) \ \ \forall s \in \mathcal{S}^{(p)}_{\text{obs}} \Big\} . \tag{S13}$$

Equivalently, $\mathcal{F}^{(p)}$ is the set of rules that agree with all local constraints imposed by the pair $(\mathbf{X}^{(p)}, \mathbf{Y}^{(p)})$.

Proposition (Pairwise degeneracy). The number of deterministic rules consistent with pair p is

$$|\mathcal{F}^{(p)}| = n^{q^{(p)}} . \tag{S14}$$

In particular, the deterministic inverse problem is uniquely identifiable from pair p *within the chosen local rule class* if and only if $q^{(p)} = 0$.

Interpretation. Equation (S14) formalizes the source of inverse ambiguity from single-pair supervision: each unobserved neighborhood type in $M^{(p)}$ leaves one unconstrained degree of freedom in the inferred

rule. Indeed, for every $s \in M^{(p)}$, the next-state value may be chosen freely from $\{0, \ldots, n-1\}$, independently across missing neighborhood types, giving $n^{q^{(p)}}$ consistent rules.

Definition (Pairwise success event). We define the event that inference returns a rule consistent on all observed neighborhoods by

$$E_p := \left\{ \hat{f}^{(p)} \in \mathcal{F}^{(p)} \right\}. \tag{S15}$$

This event captures whether the inverse model correctly fits all constraints that are actually visible in pair p.

Assumption (Pairwise inference success lower bound). Assume there exists $\alpha \in [0,1]$ such that

$$\Pr\Big(E_p \mid \mathbf{X}^{(p)}, \mathbf{Y}^{(p)} \Big) \geq 1 - \alpha, \qquad \forall p. \tag{S16}$$

This assumption describes the reliability of the inverse model on the *observed* constraints.

Definition (Near-identifying pair event). Fixing an integer $q_0 \geq 0$, we define

$$G_p := \left\{ q^{(p)} \leq q_0 \right\}, \qquad \pi := \Pr(G_p). \tag{S17}$$

The event G_p captures whether the sampled pair is *informative*: even perfect fitting on observed neighborhoods does not imply near-identification if coverage is poor, so it is useful to separate model success (E_p) from pair informativeness (G_p).

Lemma (Best-of-N_p amplification under pairwise coverage). Assuming that pair draws are independent, Equation (S16) holds for all p, and $\Pr(G_p) = \pi$, then

$$\Pr(\exists p \leq N_p : E_p \wedge G_p) \;\geq\; 1 - (1 - (1-\alpha)\pi)^{N_p}. \tag{S18}$$

Interpretation. Equation (S18) quantifies a simple but important mechanism: sampling more IC–GS2 pairs increases the probability of obtaining at least one candidate ruleset that is both (i) correct on the neighborhoods actually observed in its source pair and (ii) derived from a pair with strong neighborhood coverage. Thus, the role of larger N_p is not merely to provide more candidates, but to increase the chance that at least one candidate is sourced from a near-identifying pair.

S1.7 Orbit-Induced Coverage Proxy

The previous subsection gives a combinatorial characterization of pairwise ambiguity. We now introduce a probabilistic proxy that helps explain when high-coverage pairs are likely to arise along sampled trajectories.

Let $U \sim \mathrm{Unif}(\Lambda)$ denote a uniformly random lattice site. At a fixed coarse time t, define the neighborhood distribution

$$p_t(s) := \Pr\big(\eta_r(\mathbf{x}^t, U) = s\big), \qquad s \in \mathcal{S}. \tag{S19}$$

If pair p is sourced from time $t = t_p$, then a neighborhood type s appears at least once in the IC grid $\mathbf{x}^t$ if and only if

$$s \in \mathcal{S}_{\mathrm{obs}}^{(p)} \iff \exists u \in \Lambda : \; \eta_r(\mathbf{x}^t, u) = s. \tag{S20}$$

Using a standard independence approximation across lattice sites, we obtain the heuristic estimate

$$\Pr\Big(s \notin \mathcal{S}^{(p)}_{\text{obs}} \mid t\Big) \approx (1 - p_t(s))^{|\Lambda|}. \tag{S21}$$

Under this approximation, the expected number of missing neighborhood types in a pair sampled at time t is

$$\mathbb{E}\Big[q^{(p)} \mid t\Big] = \sum_{s \in \mathcal{S}} \Pr\Big(s \notin \mathcal{S}^{(p)}_{\text{obs}} \mid t\Big) \approx \sum_{s \in \mathcal{S}} (1 - p_t(s))^{|\Lambda|}. \tag{S22}$$

Interpretation. Equation (S22) should be interpreted as a tractable *coverage proxy*, not as an exact identity. It explains why longer or more diverse trajectories, richer IC ensembles, or larger grids can substantially reduce pairwise degeneracy in Equation (S14): all else equal, such conditions increase the probability that more neighborhood types are observed at least once. In practice, this proxy helps rationalize why inverse identifiability can improve even when the inverse model itself is unchanged.

S1.8 Local Surrogates for Coarse-Grained Non-CA Trajectories

We now leave the exact deterministic-CA setting of Equation (S12) and consider the more general case in which $\{\mathbf{x}^t\}$ arises from an arbitrary non-CA system after discretization via Equation (S1). In this regime, the relevant question is no longer whether a unique "true CA rule" exists, but whether the coarse-grained dynamics admit a useful *local surrogate* at the chosen scale.

Let $(\mathbf{X}, \mathbf{Y})$ denote a generic IC–GS2 pair drawn from the sampling procedure in Section S1.3, and let $U \sim \text{Unif}(\Lambda)$. To avoid confusion with the lattice side length N, we define the random neighborhood variable

$$Z := \eta_r(\mathbf{X}, U) \in \mathcal{S}, \qquad Y := \mathbf{Y}_U \in \{0, \dots, n-1\}. \tag{S23}$$

Here, Z is the neighborhood type observed at a uniformly random site in the IC, and Y is the corresponding next-state value at that site in GS2.

Assumption (Local-Markov-at-scale). Assume that, at the chosen coarse-graining and radius r, the next-state distribution depends approximately only on the local neighborhood:

$$\Pr(Y = y \mid \mathbf{X}, U) \approx \Pr(Y = y \mid Z) \qquad \forall y \in \{0, \dots, n-1\}. \tag{S24}$$

Here, the symbol "$\approx$" is meant informally: it indicates that the conditional law of Y is well-approximated by a function of the local neighborhood alone at the chosen coarse-graining scale. This is a conditional modeling assumption rather than a universal property. It specifies the regime in which a local CA-style surrogate is a meaningful target for the discretized system.

We then define the local conditional distribution as

$$q^\star(y \mid s) := \Pr(Y = y \mid Z = s), \qquad s \in \mathcal{S}. \tag{S25}$$

Under Equation (S24), the best achievable one-step predictor at radius r is necessarily a function of the neighborhood type $s \in \mathcal{S}$. If the chosen radius is too small relative to the effective interaction scale, then $q^\star(\cdot \mid s)$ will generally exhibit irreducible uncertainty (high conditional entropy), which places a principled ceiling on one-step prediction accuracy; this ceiling is made explicit in Section S1.10.

S1.9 Cross-Entropy Learning of Local Transition Distributions

We now define a learning objective for predicting next-state distributions from local neighborhoods. Suppose our inverse model outputs, for each neighborhood $s \in \mathcal{S}$, a categorical distribution $q_\theta(\cdot \mid s)$ on $\{0, \ldots, n-1\}$, where θ denotes the model parameters.

For a single local example with observed neighborhood s and true next-state value y, the cross-entropy loss is

$$\ell_{CE}(\theta; s, y) := -\log q_\theta(y \mid s). \tag{S26}$$

This quantity is small when the model assigns high probability to the correct next state, and large when it assigns low probability.

In our setting, local examples are obtained by first sampling an IC–GS2 pair $(\mathbf{X}, \mathbf{Y})$ and then sampling a lattice site $U \sim \mathrm{Unif}(\Lambda)$. The corresponding local input-output pair is

$$Z := \eta_r(\mathbf{X}, U), \qquad Y := \mathbf{Y}_U,$$

as defined in Equation (S23). Averaging the local loss (Equation (S26)) over this sampling procedure yields the population cross-entropy risk:

$$\mathcal{L}_{CE}(\theta) := \mathbb{E}_{(\mathbf{X},\mathbf{Y})}\, \mathbb{E}_{U\sim\mathrm{Unif}(\Lambda)} \left[-\log q_\theta(\mathbf{Y}_U \mid \eta_r(\mathbf{X}, U))\right]. \tag{S27}$$

Equivalently,

$$\mathcal{L}_{CE}(\theta) = \mathbb{E}[-\log q_\theta(Y \mid Z)],$$

since $Z = \eta_r(\mathbf{X}, U)$ and $Y = \mathbf{Y}_U$ by definition.

For CA-generated data with a ground-truth deterministic rules matrix, the corresponding local conditional distribution $q^\star(\cdot \mid s)$ from Equation (S25) is one-hot for each neighborhood type s. In that setting, minimizing Equation (S27) corresponds to aligning the inferred rules-matrix columns with the ground-truth categorical transition distributions. In the more general non-CA setting, however, $q^\star(\cdot \mid s)$ need not be one-hot. Thus, the role of cross-entropy here is to characterize the optimal local predictive target at the chosen coarse-graining and surrogate neighborhood class, rather than to imply that the underlying system is itself governed by an exact deterministic CA rule.

Proposition (Cross-entropy identification of the local conditional). Assume the model class is well-specified in the sense that there exists θ such that $q_\theta(\cdot \mid s) = q^\star(\cdot \mid s)$ for all s with $\Pr(Z = s) > 0$. If $\theta^\star \in \arg\min_\theta \mathcal{L}_{CE}(\theta)$, then

$$q_{\theta^\star}(y \mid s) = q^\star(y \mid s) \quad \forall s \text{ with } \Pr(Z = s) > 0,\ \forall y. \tag{S28}$$

Interpretation. Equation (S28) clarifies the target of learning in the non-CA setting: population cross-entropy minimization identifies the optimal *local conditional surrogate* induced by the chosen coarse-graining and surrogate neighborhood class. It does *not* claim recovery of a unique "true CA rule" for an arbitrary system. As in the deterministic setting, neighborhood types with $\Pr(Z = s) = 0$ remain unconstrained by the population objective.

S1.10 Deterministic Local Surrogate and Optimal One-Step Accuracy

We now pass from a probabilistic local surrogate to its best deterministic approximation. Define the deterministic surrogate rule (i.e., the Bayes classifier over neighborhood types) by

$$f^{\dagger}(s) := \arg\max_{y\in\{0,\dots,n-1\}} q^{\star}(y \mid s). \tag{S29}$$

We also define the population one-step accuracy of a deterministic rule by

$$A(f) := \Pr(f(Z) = Y). \tag{S30}$$

Corollary (Optimal deterministic one-step accuracy). We have

$$f^{\dagger} \in \arg\max_{f} A(f), \qquad A_r^{\star} := A(f^{\dagger}) = \sum_{s\in\mathcal{S}} \Pr(Z = s) \max_{y} q^{\star}(y \mid s). \tag{S31}$$

Interpretation. Equation (S31) gives the best possible one-step accuracy attainable by any *deterministic* local rule within the chosen hypothesis class. In particular, $A_r^{\star}$ is a radius-dependent approximation ceiling: changing r changes the neighborhood space $\mathcal{S}$ in Equation (S2), and therefore changes the best achievable local surrogate. Increasing r may improve achievable accuracy by resolving hidden dependencies, but it also enlarges the neighborhood space and typically increases the data requirements for accurate inference.

S1.11 Selection Guarantee for Self-Scoring Best-of-N_p Pairwise Inferences

The preceding subsections addressed identifiability and approximation at the level of the underlying inverse problem. We now formalize the practical ensemble-selection heuristic used in the experiments detailed later on in Section S2: generating multiple pairwise candidate rules and retaining the one with the highest self-score.

For each pair p, define the self-score

$$S_p := \widehat{A}_p\left(\hat{f}^{(p)}\right) \in [0, 1], \tag{S32}$$

and define the maximizing index

$$p^{\star} := \arg\max_{1\le p\le N_p} S_p, \tag{S33}$$

so that $\hat{f}_{\text{best}} = \hat{f}^{(p^{\star})}$.

Definition (High-self-score event). Fix $\tau \in [0, 1]$. We define

$$H_p(\tau) := \{S_p \ge \tau\}. \tag{S34}$$

Assumption (Per-pair high-score probability lower bound). Assume that for some $\rho(\tau) \in [0, 1]$,

$$\Pr(H_p(\tau)) \ge \rho(\tau) \qquad \forall p, \tag{S35}$$

where the probability is taken over randomness in pair sampling (choice of t_p, IC ensemble, etc.) and any stochasticity in Infer.

Theorem (Best-of-N_p amplification under self-scoring). Assume that pair draws are independent and that Equation (S35) holds for all p. Then

$$\Pr\left(\max_{1\le p\le N_p} S_p \ge \tau\right) = \Pr\left(\bigcup_{p=1}^{N_p} H_p(\tau)\right) \ge 1-(1-\rho(\tau))^{N_p}. \tag{S36}$$

Interpretation. Equation (S36) formalizes the intuition behind best-of-N_p selection: even if a single pair has only moderate probability of producing a high-self-score candidate, the chance that *at least one* of N_p independently sampled pairs does so increases rapidly with N_p. This result does not guarantee out-of-sample generalization; rather, it justifies the practical strategy of generating multiple pairwise candidates and retaining the one most consistent with its own source pair.

Deterministic CA special case. Under the deterministic CA generator assumption given by Equation (S12), if $\hat{f}^{(p)} \in \mathcal{F}^{(p)}$ (cf. Equation (S13)), then $S_p = 1$ for that specific source pair. Thus, with E_p defined in Equation (S15),

$$\Pr\left(\max_{p\le N_p} S_p = 1\right) \ge \Pr\left(\bigcup_{p=1}^{N_p} E_p\right). \tag{S37}$$

If, in addition, pair draws are independent and $\Pr(E_p) \ge 1-\alpha$ for all p, then

$$\Pr\left(\max_{p\le N_p} S_p = 1\right) \ge 1-\alpha^{N_p}. \tag{S38}$$

Remark (Relation to coverage-conditioned amplification). The self-scoring amplification result above is intentionally agnostic to the source of pair quality. By contrast, Equation (S18) in Section S1.6 isolates one specific mechanism: the joint occurrence of model success on observed constraints (E_p) and strong neighborhood coverage (G_p). Thus, the earlier result explains *why* certain pairwise candidates are especially valuable in the exact deterministic-CA setting, whereas the present subsection explains *why* best-of-N_p self-selection is an effective practical heuristic more generally.

S1.12 Why Self-Scoring Selection Is Preferable to Naive Rule Averaging in the Pairwise-Degenerate Regime

We finally compare the self-scoring ensemble-selection heuristic in Section S1.11 to a natural alternative in the binary deterministic setting: averaging inferred rules across the ensemble and then rounding each neighborhood-type assignment to the nearest deterministic value.

Assume throughout this subsection that the state space is binary, so each deterministic local rule has codomain $\{0,1\}$. Let $\mathcal{S} = \{s_1,\ldots,s_m\}$ denote the set of neighborhood types, where $m := |\mathcal{S}|$. For each pair p, the inferred rule $\hat{f}^{(p)} : \mathcal{S} \to \{0,1\}$ may be written equivalently as the binary vector

$$\mathbf{r}^{(p)} := \left(\hat{f}^{(p)}(s_1),\ldots,\hat{f}^{(p)}(s_m)\right) \in \{0,1\}^m. \tag{S39}$$

A naive ensemble-average rule may then be defined coordinatewise by majority vote:

$$\bar{f}(s_j) := \mathbf{1}\left\{\frac{1}{N_p}\sum_{p=1}^{N_p} \hat{f}^{(p)}(s_j) \ge \frac{1}{2}\right\}, \qquad j=1,\ldots,m, \tag{S40}$$

with a fixed tie-breaking convention if needed. Thus, $\bar{f}$ is obtained by averaging the inferred assignments for each neighborhood type across the ensemble and rounding back to a deterministic binary rule.

While Equation (S40) is natural, it is not generally well matched to the pairwise inverse problem studied here. The reason is that the inferred rules $\{\hat{f}^{(p)}\}_{p=1}^{N_p}$ are not independent estimates of a single fully constrained target rule. Rather, each $\hat{f}^{(p)}$ is inferred from a different IC–GS2 pair and is therefore directly constrained only on the neighborhood types observed in its own source pair, namely $\mathcal{S}_{\text{obs}}^{(p)}$. For neighborhood types outside $\mathcal{S}_{\text{obs}}^{(p)}$, the inferred assignments may remain weakly constrained, degenerate, or effectively arbitrary. Coordinatewise averaging can therefore produce a hybrid rule that need not be especially consistent with any actual pair in the ensemble.

To formalize this, we consider the following stylized regime.

Assumption (One coherent high-quality candidate plus heterogeneous unconstrained assignments). Assume there exists an index $p^\star$ such that:

1. $\hat{f}^{(p^\star)}$ is a high-quality candidate inferred from an informative pair, in the sense that it attains the highest self-score in the ensemble and is reliable on many neighborhood types relevant to its source pair;
2. for a neighborhood type s_j that is constrained by pair $p^\star$ but unobserved for most other pairs, the values $\{\hat{f}^{(p)}(s_j)\}_{p\neq p^\star}$ behave approximately as independent Bernoulli(1/2) variables.

The second condition is not intended as a universal law; it idealizes the pairwise-degenerate regime in which many inferred rules agree on neighborhood types they observe but vary idiosyncratically on neighborhood types they do not observe.

Proposition (Averaging dilutes isolated informative assignments). Assume N_p is odd, so that the majority vote in Equation (S40) has no exact tie. Under the above assumption, for any neighborhood type s_j that is informative for $p^\star$ but effectively unconstrained for the remaining $N_p - 1$ candidates, the probability that the averaged rule agrees with the high-quality candidate on that neighborhood type is

$$\Pr\Big(\bar{f}(s_j) = \hat{f}^{(p^\star)}(s_j)\Big) = \frac{1}{2} + \frac{1}{2}\binom{N_p - 1}{\frac{N_p-1}{2}} 2^{-(N_p-1)}. \tag{S41}$$

In particular,

$$\Pr\Big(\bar{f}(s_j) = \hat{f}^{(p^\star)}(s_j)\Big) \longrightarrow \frac{1}{2} \qquad \text{as } N_p \to \infty. \tag{S42}$$

Interpretation. Equation (S41) shows that, on neighborhood types for which only one candidate carries reliable information while the rest are effectively unconstrained, coordinatewise averaging is only slightly better than a coin flip, and this advantage vanishes as the ensemble grows. Intuitively, averaging does not preserve the coherence of a good inferred rule; instead, it mixes together neighborhood-type assignments that were inferred under different pairwise constraints, producing a hybrid rule assembled from mismatched pieces.

By contrast, the self-scoring rule

$$\hat{f}_{\text{best}} = \hat{f}^{(p^\star)} \quad \text{whenever} \quad p^\star = \arg\max_{1\le p\le N_p} S_p$$

retains an entire candidate rule inferred from a single pair. Thus, whenever the ensemble contains at least one genuinely high-quality candidate, self-scoring selection preserves that coherent rule in its entirety, whereas naive averaging can dilute or overwrite its informative assignments on neighborhood types that are poorly covered across the rest of the ensemble.

Relation to the amplification results above. The advantage of self-scoring is therefore structural, not merely numerical. Sections S1.6 and S1.11 show that increasing N_p raises the probability that the ensemble contains at least one candidate inferred from a successful and informative pair. The present subsection adds that, once such a candidate exists, selecting it by self-score is generally more principled than coordinatewise averaging in the pairwise-degenerate regime, because averaging does not respect the pairwise origin of the inferred constraints.

Conclusion. We therefore view the sample-best self-scoring heuristic as better matched to the inverse problem studied here than naive rule averaging. The latter can work well only when neighborhood-type assignments are stable across the ensemble, which is precisely the condition that fails in the presence of strong pairwise degeneracy. In contrast, self-scoring is designed to identify and preserve a single coherent high-quality rule sourced from an informative IC–GS2 pair.

S1.13 Connection Between Local Surrogate Accuracy and Macroscopic Observables

The preceding subsections characterize inference and approximation at the level of one-step local prediction. We now briefly connect that framework to the macroscopic observables considered later in the Allen–Cahn study. The goal of this subsection is not to prove that accurate local surrogate rules necessarily reproduce all large-scale behavior, but rather to clarify a simple conditional principle: if a macroscopic observable is sufficiently stable to small configuration errors, then a high-accuracy local surrogate can also yield a small one-step error in that observable.

Let $\Phi : \{0, \ldots, n-1\}^{\Lambda} \to \mathbb{R}$ denote a scalar observable of a lattice configuration. For a deterministic rule f, consider the one-step observable discrepancy

$$\Delta_{\Phi}(f) := \left|\Phi(\mathcal{C}_f(\mathbf{X})) - \Phi(\mathbf{Y})\right|, \tag{S43}$$

where $(\mathbf{X}, \mathbf{Y})$ denotes a generic IC–GS2 pair as in Section S1.3. This quantity measures how much the observable computed from the surrogate one-step prediction differs from the observable computed from the ground-truth next state.

To relate Equation (S43) to state-level prediction error, we introduce a stability assumption on the observable itself.

Assumption (Observable stability under Hamming perturbations). Assume there exists a constant $L_\Phi \geq 0$ such that for all configurations $\mathbf{a}, \mathbf{b} \in \{0, \ldots, n-1\}^{\Lambda}$,

$$|\Phi(\mathbf{a}) - \Phi(\mathbf{b})| \leq L_\Phi \frac{1}{|\Lambda|} \sum_{u \in \Lambda} \mathbf{1}\{a_u \neq b_u\}. \tag{S44}$$

That is, the observable changes by at most a constant multiple of the normalized Hamming distance between the two configurations.

Proposition (One-step observable error bound). Under Equation (S44), for any deterministic rule f,

$$\Delta_\Phi(f) \leq L_\Phi\big(1 - \widehat{A}_p(f)\big) \tag{S45}$$

when $(\mathbf{X}, \mathbf{Y}) = (\mathbf{X}^{(p)}, \mathbf{Y}^{(p)})$ is a sampled pair and $\widehat{A}_p(f)$ is the pairwise forward accuracy defined in Equation (S9).

Interpretation. Equation (S45) states that if Φ is stable in the sense of Equation (S44), then a rule with high one-step state accuracy on a given pair must also have small one-step error in that observable on the same pair. Thus, for observables that are insensitive to a small fraction of cellwise discrepancies, accurate local surrogate prediction can translate into accurate one-step macroscopic prediction.
This connection is deliberately conditional. First, the constant L_Φ depends on the observable: some observables are much more sensitive than others to local configuration errors. Second, Equation (S45) is a one-step statement. Even if the one-step observable discrepancy is small, repeated recursive application of a surrogate rule can accumulate state errors over time, and those accumulated errors may eventually produce substantial macroscopic divergence. Third, for non-CA systems this bound does not remove the approximation ceiling identified in Section S1.10; if the chosen coarse-graining and radius r do not admit a sufficiently accurate local surrogate, then corresponding macroscopic agreement may also be limited.

Relevance to the Allen–Cahn study. The macroscopic quantities examined later in this Supporting Information are intended precisely as coarse observables of this kind: they summarize large-scale structural features of the evolving discretized field rather than requiring exact cellwise agreement everywhere. The argument above therefore provides a rationale for why a reasonably accurate one-step local surrogate may still reproduce certain short-horizon macroscopic trends of a coarse-grained non-CA system, even when exact long-horizon trajectory matching is not achieved.

S2 Approximating Allen–Cahn Dynamics with a Cellular Automaton

S2.1 The Allen–Cahn System

The process of phase separation in multi-component alloy systems has been broadly described by the Allen–Cahn equation, which may be conceptualized as the L^2-gradient flow of the Ginzburg-Landau energy functional [1], which may be defined as:

$$E_{GL}[u] := \int_\Omega \left(\frac{D}{2} |\nabla u(\mathbf{x})|^2 + \frac{1}{\varepsilon^2} W(u) \right) d\mathbf{x}. \tag{S46}$$

where $u(\mathbf{x})$ is a function of a spatial coordinate ($\mathbf{x} = (x, y) \in \Omega \subset \mathbb{R}^2$) representing a phase field (otherwise known as an order parameter), for which values close to -1 or 1 in a particular region correspond to strong single-phase ordering, and values close to 0 correspond to phase boundaries; Ω is the spatial domain on which the phase field is defined; D is a diffusion coefficient; ε is an interface thickness parameter; and $W(u)$ is a double well potential function, defined as follows:

$$W(u) := \frac{1}{4}(u^2 - 1)^2; \quad W : \mathbb{R} \to \mathbb{R}. \tag{S47}$$

The L^2-gradient flow associated with the Ginzburg-Landau energy $E_{GL}[u]$ yields the Allen–Cahn dynamics [2, 3]:

$$\partial_t u = -\frac{\delta E_{GL}}{\delta u} = D\nabla^2 u - \frac{1}{\varepsilon^2} W'(u) = D\nabla^2 u + \frac{1}{\varepsilon^2}(u - u^3). \tag{S48}$$

This reaction-diffusion partial differential equation (PDE) drives the phase field toward the stable bulk phases and penalizes interfaces via the Laplacian term.
To investigate the capability of AutomataGPT (the $N_{RM} = 100$ version) to infer binary CA rulesets that act as local surrogate models for a discretized non-CA dynamical system, we used binarized Allen–

Cahn dynamics as a testbed for the inverse problem. The Allen–Cahn PDE was chosen due both in part to its notability and agreement with real-world phase separation behavior across a variety of physical systems [4, 5, 6]. The reason for binarization of the Allen–Cahn phase field was to account for the limited CA rule space on which AutomataGPT was trained; the rule space only contained the rulesets of binary CA systems. A flowchart of the following experiment is provided in Figure S1.

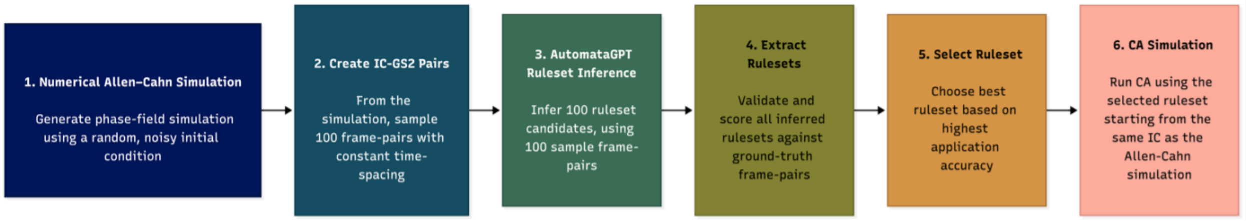

Figure 1: Flowchart illustrating the process of inferring and applying a CA ruleset to approximate Allen–Cahn phase separation dynamics.

S2.2 Ruleset Inference Procedure

S2.2.1 Allen–Cahn Data Generation

Generating a Random Initial Condition. To initialize Allen–Cahn phase field simulations, we always used a noisy, random initial condition (IC). The IC, denoted as $u_0 \in \mathbb{R}^{N\times N}$, is a 2D array constructed as follows.

First Gaussian noise is generated for every cell in the IC grid:

$$\eta_{i,j} \sim \mathcal{N}(0,1) \quad \text{independently} \quad \forall i,j \in \{1,\ldots,N\}. \tag{S49}$$

Next, the spatial mean of the noise is computed:

$$\bar{\eta} = \frac{1}{N^2}\sum_{i=1}^{N}\sum_{j=1}^{N}\eta_{i,j}. \tag{S50}$$

Each noise entry is then mean-centered and scaled by a preselected noise amplitude parameter (σ, set to 0.1 in all our experiments):

$$\nu_{i,j} = \sigma(\eta_{i,j} - \bar{\eta}). \tag{S51}$$

Finally, each element of u_0, $u^0_{i,j}$, is defined as follows:

$$u^0_{i,j} = \text{clip}_{[-1,1]}(\nu_{i,j}), \tag{S52}$$

where the clip function is defined such that:

$$\text{clip}_{[-1,1]}(z) = \min(1, \max(-1, z)). \tag{S53}$$

The Discrete Phase Field. To numerically compute Allen–Cahn trajectories, we discretized the phase field $u(\mathbf{x}, t)$ on a uniform $N \times N$ Cartesian grid with spacing Δx, and evolved the system forward in time using an explicit finite-difference scheme with periodic boundary conditions. The Laplacian term in Equation (S48) was approximated using a standard five-point stencil, yielding the update rule:

$$u_{i,j}^{n+1} = u_{i,j}^{n} + \Delta t \left[D(\nabla^2 u^n)_{i,j} + \frac{1}{\varepsilon^2} \left(u_{i,j}^{n} - (u_{i,j}^{n})^3 \right) \right], \quad \text{(S54)}$$

where $u_{i,j}^n$ denotes the phase-field value at grid index (i, j) and timestep n; D and ε once again refer to the diffusion coefficient and interface thickness parameter respectively; and $(\nabla^2 u^n)_{i,j}$ is the discrete Laplacian. The discrete Laplacian may be written as its five-point stencil [7] formulation:

$$(\nabla^2 u^n)_{i,j} \approx \frac{u_{i+1,j}^n + u_{i-1,j}^n + u_{i,j+1}^n + u_{i,j-1}^n - 4u_{i,j}^n}{\Delta x^2}, \quad \text{(S55)}$$

where all indices are taken modulo N to enforce periodic boundary conditions (toroidal grid).

The Discrete Timestep Parameter. To ensure stable evolution across parameter regimes, we selected the timestep Δt based on a characteristic timescale τ defined by the minimum of a diffusion timescale and a reaction timescale:

$$\tau = \min\left(\frac{\Delta x^2}{D}, \varepsilon^2 \right), \quad \text{(S56)}$$

$$\Delta t = dt^* \cdot \tau, \quad \text{(S57)}$$

where dt^* is a dimensionless timestep parameter. To ensure consistency, we used $(D, \varepsilon, \Delta x, dt^*) = (1.0, 0.1, 0.1, 0.1)$ for all Allen–Cahn simulations.

S2.2.2 Data Preprocessing

Binarization. Since AutomataGPT infers rulesets for binary CA, we converted continuous Allen–Cahn phase field snapshots $u(\mathbf{x}, t)$ into binary fields $b(\mathbf{x}, t) \in \{0, 1\}$ using thresholding as follows:

$$\forall \mathbf{x} \in \Omega,\ \forall t, \quad b(\mathbf{x}, t) = \begin{cases} 0 & \text{for } u(\mathbf{x}, t) \le \theta(t), \\ 1 & \text{for } u(\mathbf{x}, t) > \theta(t). \end{cases} \quad \text{(S58)}$$

where $\theta(t)$ is defined as the median value across the entire discretized phase field at a given time t. Discretization is a prerequisite for binarization and is described in the following section.

Space-time Discretization. We let $u(\mathbf{x}, t)$ continue to denote the continuous Allen–Cahn field defined over the spatial domain $\Omega \subset \mathbb{R}^2$. To allow for numerical simulation as previously described, we discretize Ω on a uniform $N \times N$ Cartesian grid with spacing Δx, and let $\mathbf{x}_{i,j} \in \Omega$ denote the spatial location of grid index (i, j) for $i, j = 1, \ldots, N$. For convenience, we define the discrete phase field at solver timestep n (total time elapsed $= n\Delta t$) as the field-valued object:

$$u^n := \{u_{i,j}^n\}_{i,j=1}^N \in \mathbb{R}^{N \times N} = \begin{pmatrix} u_{1,1}^n & \cdots & u_{1,N}^n \\ \vdots & \ddots & \vdots \\ u_{N,1}^n & \cdots & u_{N,N}^n \end{pmatrix}, \quad \text{(S59)}$$

where $u_{i,j}^n := u(\mathbf{x}_{i,j}, n\Delta t)$. Using u^n, a median value may be determined for binarization thresholding (i.e. the median of the set of all entries of the matrix u^n), and the spatially discrete binarized phase field defined as follows:

$$\mathbf{b}^n := \{b^n_{i,j}\}_{i,j=1}^N \in \{0,1\}^{N\times N} = \begin{pmatrix} b^n_{1,1} & \cdots & b^n_{1,N} \\ \vdots & \ddots & \vdots \\ b^n_{N,1} & \cdots & b^n_{N,N} \end{pmatrix}. \tag{S60}$$

In addition to spatial discretization, we introduce a temporal discretization that specifies the spacing between successive samples of the discretized regular and binarized phase-field trajectories in solver time. Letting $\mathbf{u}^n \in \mathbb{R}^{N\times N}$ and $\mathbf{b}^n \in \{0,1\}^{N\times N}$ denote the discretized regular and binarized phase fields at solver timestep n, corresponding to physical time $t = n\Delta t$, we introduce an integer time spacing $s \in \mathbb{N}$, which defines the number of Allen–Cahn solver timesteps separating successive observations of the system. Using this spacing, we define a temporally coarse-grained binary trajectory by sampling the binarized phase field every s solver steps,

$$\mathbf{b}^k := \mathbf{b}^{ks}, \quad k = 0, 1, 2, \ldots \tag{S61}$$

where the index k acts as a label for discrete time in the resulting sequence ($k = 0$ corresponds to the initial condition). The physical time associated with coarse timestep k is given by:

$$t^k_{\text{phys}} = k\, s\, \Delta t. \tag{S62}$$

The sequence $\{\mathbf{b}^k\}_{k=k_0}^{k_F}$ thus represents a temporally discretized evolution of the binarized phase field at resolution s from coarse-grained time index k_0 to k_F, and serves as the discrete-time representation of the underlying continuous dynamics used in subsequent analysis and modeling. This temporal discretization constitutes a coarse-graining of the phase-field evolution and does not modify the solver timestep Δt or the spatial resolution Δx.

We explored multiple values of the temporal spacing parameter s and found that the qualitative agreement between the binarized Allen–Cahn trajectories and their CA representations depended strongly on the chosen temporal resolution. Based on our exploratory analysis, we fixed $s = 20$ for the experiments presented here.

S2.2.3 Generating an Ensemble of Inferences

To ensure we have exactly 100 pairs, we numerically compute a binarized Allen–Cahn trajectory $\{\mathbf{b}^k\}_{k=0}^{100}$ using $k_F s = 100 \times 20 = 2000$ numerical steps. Given the temporally coarse-grained binary trajectory at time spacing $s = 20$ (see Equation (S56) and Equation (S57)), we construct an ensemble of inverse-problem inputs by sampling multiple "IC-GS2" pairs from $\{\mathbf{b}^k\}_{k=0}^{100}$. We form pairs $(\mathbf{b}^k, \mathbf{b}^{k+1})\ \forall k \in \{0, 1, 2, \ldots, 98, 99\}$, where $\mathbf{b}^k$ is treated as the IC and $\mathbf{b}^{k+1}$ as the ground-truth one-step future—"game state 2" (GS2). These pairs are serialized into the AutomataGPT inverse prompt format and passed through the model to generate candidate RMs. To account for inference variability and reduce sensitivity to any single samples pair, we generate an ensemble of 100 candidate RMs by repeating the inference procedure across the 100 IC-GS2 pairs. Thus, ruleset inference may be formalized as follows:

$$\{RM_k\}_{k=0}^{99} := \{\mathcal{I}_{\text{AGPT}}(\mathbf{b}^k, \mathbf{b}^{k+1})\}_{k=0}^{99}, \tag{S63}$$

where we define $\mathcal{I}_{\text{AGPT}}$ as the AutomataGPT inverse problem inference operator, which maps an IC-GS2 pair to a corresponding RM. Since each ensemble element corresponds to an IC-GS2 pair sampled at time index k along the same Allen–Cahn trajectory, we index the inferred rulesets by the same k. Each output from AutomataGPT is passed into a RM retained only if it satisfied basic validity constraints (Section S2.1.4).

S2.2.4 Distilling the Best Ruleset from the Ensemble

Each inferred ruleset is represented as a 2D binary non-directional deterministic CA rules matrix (RM) mapping local neighborhoods (metastates) to cell states. We discard malformed (illogical) outputs and retain only valid RMs (those with correct dimensions and with columns corresponding to valid categorical distributions over next states).
To compare the rulesets, we evaluate each candidate rule RM_k on its corresponding IC-GS2 pair ($\mathbf{b}^k$ and $\mathbf{b}^{k+1}$). Applying RM_k on $\mathbf{b}^k$ yields a CA prediction of the form:

$$\hat{\mathbf{b}}^{k+1} = \mathcal{C}(\mathbf{b}^k, RM_k), \tag{S64}$$

where $\hat{\mathbf{b}}^{k+1}$ is the computed GS2 in accordance with the inferred ruleset RM_k, and $\mathcal{C}$ is the forward CA update operator, which takes in an IC and RM and outputs the corresponding GS2.
We quantify ruleset quality using the inferred rules matrix application accuracy (A_{IRMA}), as defined by Equation 4 within the main text. Specifically, A_{IRMA} measures the cell-wise agreement between the ground-truth GS2 ($\mathbf{b}^{k+1}$) and the new GS2 ($\hat{\mathbf{b}}^{k+1}$) obtained by applying an inferred ruleset (RM_k) to its corresponding IC ($\mathbf{b}^k$). The "sample-best ruleset" RM_{best} is the RM achieving the highest inferred rules matrix application accuracy across the ensemble:

$$k_{\text{best}} = \arg\max_k A_{\text{IRMA}}\left(\mathbf{b}^{k+1}, \mathcal{C}(\mathbf{b}^k, RM_k)\right), \tag{S65}$$

$$RM_{\text{best}} = RM_{k_{\text{best}}}. \tag{S66}$$

For the purposes of approximating Allen–Cahn dynamics via CA, we exclusively use the RM_{best} rule, extracted from one ensemble of 100 IC-GS2 pairs, all from the same trajectory.

S2.3 Simulating the Cellular Automaton

S2.3.1 Applying the Best Ruleset on the Original 16 by 16 Grid

To determine how effectively RM_{best} generalized to long-time-horizon Allen–Cahn dynamics, RM_{best} was applied recursively starting from the original 16×16 binarized IC $\mathbf{b}^0$ to construct a new CA trajectory (orbit) or length 100, denoted $\{\tilde{\mathbf{c}}^k\}_{k=0}^{99}$, like so:

$$\tilde{\mathbf{c}}^{k+1} = \mathcal{C}(\tilde{\mathbf{c}}^k, RM_{\text{best}}), \quad \text{where } \tilde{\mathbf{c}}^0 = \mathbf{b}^0. \tag{S67}$$

For a CA grid and a ground-truth binarized Allen–Cahn grid at a given time index k, the accuracy of the CA simulation $A_{\text{CA}}(k) \in [0, 1]$ may be computed using the following equation:

$$A_{\text{CA}}(k) = \frac{1}{N^2} \sum_{i=1}^{N} \sum_{j=1}^{N} \mathbf{1}\{\tilde{c}_{i,j}^k = b_{i,j}^k\}, \tag{S68}$$

where i and j are discrete-grid indices, N is the width/length of the square grid in units of Δx ($N = 16$ for the small grid).

S2.3.2 Applying the Best Ruleset on a Large 256 by 256 Grid

Overview. The goal of running a large (256×256) simulation using both the Allen–Cahn PDE and the sample-best CA ruleset was the characterize the accuracy with which the CA simulation could capture not

only local Allen–Cahn dynamics, but global dynamics as well. While the small-scale CA simulation borrowed its initial condition from the random initial condition used for the original Allen–Cahn simulation, the large-scale simulation used a new noisy IC.

Running PDE and CA Simulations. A new 256 × 256 IC was randomly generated using the method formalized by Equations S4-S8. An Allen–Cahn trajectory was numerically computed and subsequently binarized using previously described methods. Using the sample-best ruleset, a CA simulation was computed starting from the binarized version of the same IC.

Computing Macroscopic Observables. Since the goal of this experiment was to find global, large-lengthscale, long-timescale agreements and disagreements, three scalars were chosen (dominant wavenumber, k^*; correlation length, ξ; and interfacial energy, E_{int}) to characterize the overall behavior of the system at given time indices t (we previously referred to these indices using k; here we use t to avoid conflation with wavenumbers). Considering that each frame (i.e. snapshot) of the binarized Allen–Cahn simulations and CA simulations is of the form $\mathbf{b}^t := \{b^t_{i,j}\}_{i,j=1}^N \in \{0,1\}^{N\times N}$, the following procedure is used. First, we define a signed field:

$$\forall i, j \in \{1, \ldots, N\}, \quad s_{i,j}(t) := 2b^t_{i,j} - 1 \in [-1, 1]. \tag{S69}$$

Next, we compute the 2D Fourier transform (denoted as FFT2) of the signed field:

$$\hat{s}(t) = \text{FFT2}\big(\{s_{i,j}(t)\}_{i,j=1}^N\big)\,. \tag{S70}$$

This step is followed by computing the structure factor $S(t)$, where the elements are computed like so:

$$\forall p, q \in \{1, \ldots, N\}, \quad S_{p,q}(t) = |\hat{s}_{p,q}(t)|^2\,. \tag{S71}$$

Using $S(t)$, the dominant wavenumber k^* is computed via the following steps. First, the magnitude of the physical wavevector across the discretized, signed phase field is:

$$k_{p,q} := \|\mathbf{k}_{p,q}\| = \sqrt{k_x(p)^2 + k_y(q)^2}, \tag{S72}$$

where

$$\mathbf{k}_{p,q} := (k_x(p), k_y(q)) := \left(\frac{2\pi}{N\Delta x}\tilde{p},\ \frac{2\pi}{N\Delta x}\tilde{q}\right), \tag{S73}$$

and $\tilde{p}, \tilde{q}$ are wrapped frequency indices:

$$\tilde{p} := \begin{cases} p, & 0 \le p \le \lfloor \frac{N}{2} \rfloor, \\ p - N, & \lfloor \frac{N}{2} \rfloor < p \le N - 1, \end{cases} \qquad \tilde{q} := \begin{cases} q, & 0 \le q \le \lfloor \frac{N}{2} \rfloor, \\ q - N, & \lfloor \frac{N}{2} \rfloor < q \le N - 1. \end{cases} \tag{S74}$$

Next, we find the dominant mode's frequency indices, avoiding the mean mode:

$$(p^*, q^*) \in \arg \max_{(p,q)\neq(0,0)} S_{p,q}(t). \tag{S75}$$

Finally, the dominant wavenumber is defined as:

$$k^*(t) := \|\mathbf{k}_{p^*,q^*}\|. \tag{S76}$$

To find the correlation length ξ, we first computed the 2D autocorrelation using the Inverse 2D Fast-Fourier Transform (IFFT2) of the structure factor $S(t)$,

$$C := \text{IFFT2}(S), \tag{S77}$$

(where for conciseness $S(t) = S$) and then shifted C using `fftshift` to center the zero-displacement peak in the array,

$$C_{\text{shift}} := \text{fftshift}(C). \tag{S78}$$

Next, we normalized the array such that the peak value was 1:

$$\bar{C} := \frac{C_{\text{shift}}}{\max_{i,j} C_{\text{shift},i,j}}. \tag{S79}$$

Now defining the distance from the center of each grid cell as:

$$r_{i,j} = \Delta x \sqrt{\left(i - \left\lfloor \frac{N}{2} \right\rfloor\right)^2 + \left(j - \left\lfloor \frac{N}{2} \right\rfloor\right)^2}, \tag{S80}$$

the correlation length is the smallest such $r_{i,j}$ where $\bar{C}_{i,j}$ falls below $\frac{1}{e}$:

$$\xi(t) = \min\left(r_{i,j} : \ \bar{C}_{i,j} \leq \frac{1}{e}\right). \tag{S81}$$

Lastly, the interfacial energy (which is approximated here as proportional to the interface length for a binary phase field) is defined as:

$$E_{\text{int}}(t) = E_x(t) + E_y(t), \tag{S82}$$

where $E_x(t)$ and $E_y(t)$ are the components for each interface direction:

$$E_x(t) = \sum_{i=1}^{N} \sum_{j=1}^{N} \left| b_{i,j}^t - b_{i,(j+1) \bmod N}^t \right|, \tag{S83}$$

$$E_y(t) = \sum_{i=1}^{N} \sum_{j=1}^{N} \left| b_{i,j}^t - b_{(i+1) \bmod N,j}^t \right|. \tag{S84}$$

S2.3.3 Applying the Best Ruleset on a Droplet Shrinkage Simulation

Overview. A final experiment was performed in which a numerical Allen–Cahn simulation was computed (and binarized), as well as a CA simulation using the same sample-best ruleset as previously, using a series of ICs containing circular "droplets" of a single phase with varying radii "immersed" in the other phase. Under classical Allen–Cahn dynamics, small droplets are expected to shrink until they disappear due to the high curvature penalty. This experiment was conducted to determine at which scale (relative to the individual grid cell size) the CA system exhibits "pinning," a phenomenon where dynamics freeze due to grid size effects reducing local curvature beyond a critical threshold. Since the methods for running numerical PDE and CA simulations have been previously described, the following section will focus solely on formalizing the ICs for this experiment.

Defining Initial Conditions. We define a set of nominal initial droplet radii (in units of Δx):

$$R_{\text{set}} = \{1, 2, 3, 4, 6, 8, 10, 12, 14, 16, 18, 20\}.$$

For each nominal initial radius r_0 in R_{set}, we construct a circular phase-field initial condition u_0^{circ} on a $N \times N$ grid, centered at a fixed grid point $\mathbf{c} = (c_x, c_y) = \left(\left\lfloor \frac{N}{2} \right\rfloor, \left\lfloor \frac{N}{2} \right\rfloor\right)$. For each grid cell (i, j), define the distance to the center as:

$$r(i, j) = \sqrt{(i - c_x)^2 + (j - c_y)^2}. \tag{S85}$$

We set the inner and the outer phase accordingly: $u_{\text{in}} = +1$ and $u_{\text{out}} = -1$. Next, using a smoothed interface (tanh profile) with interface width $w = 2\Delta x$, the initial phase field is:

$$u_0^{\text{circ}}(i, j) = u_{\text{out}} + (u_{\text{in}} - u_{\text{out}}) \cdot 0.5 \cdot \left(1 - \tanh\left(\frac{r(i, j) - r_0}{w}\right)\right). \tag{S86}$$

A Note on Binarization. For this experiment, binarization of Allen–Cahn trajectories was achieved using the same method as previously described with one change: the value for θ in Equation (S58) was explicitly set to 0, instead of being assigned to the median field value. This was done because the median phase field value was not close to 0 for most ICs, unlike the other experiments.

S3 Results and Discussion

S3.1 Small-scale Simulation

In the small-scale simulation (based off the same Allen–Cahn trajectory as was used to infer the sample-best CA ruleset) reasonable agreement was observed both qualitatively (Figure S2) and quantitatively (Figure S3), though dynamics were far from perfect agreement. In Figure S2, the formation of distinct clusters of black cells at times 1 and 2 (in units of $20\Delta t$) was observed in both simulation types. Likewise, overall phase field homogenization and phase cluster edge smoothing were similarly observed in both simulations at times 3,4, and 5. However, while at later times the binarized Allen–Cahn phase field continued to smooth out phase interfaces, gradually eliminating any curvature, the CA simulation got “locked” in place, with some phase interface curvature remaining at time 20. The CA approximation’s considerable deviation from ground truth after time > 4 was due in part to compounding errors at every timestep. We discuss other causes for disagreement between the simulations in later sections.

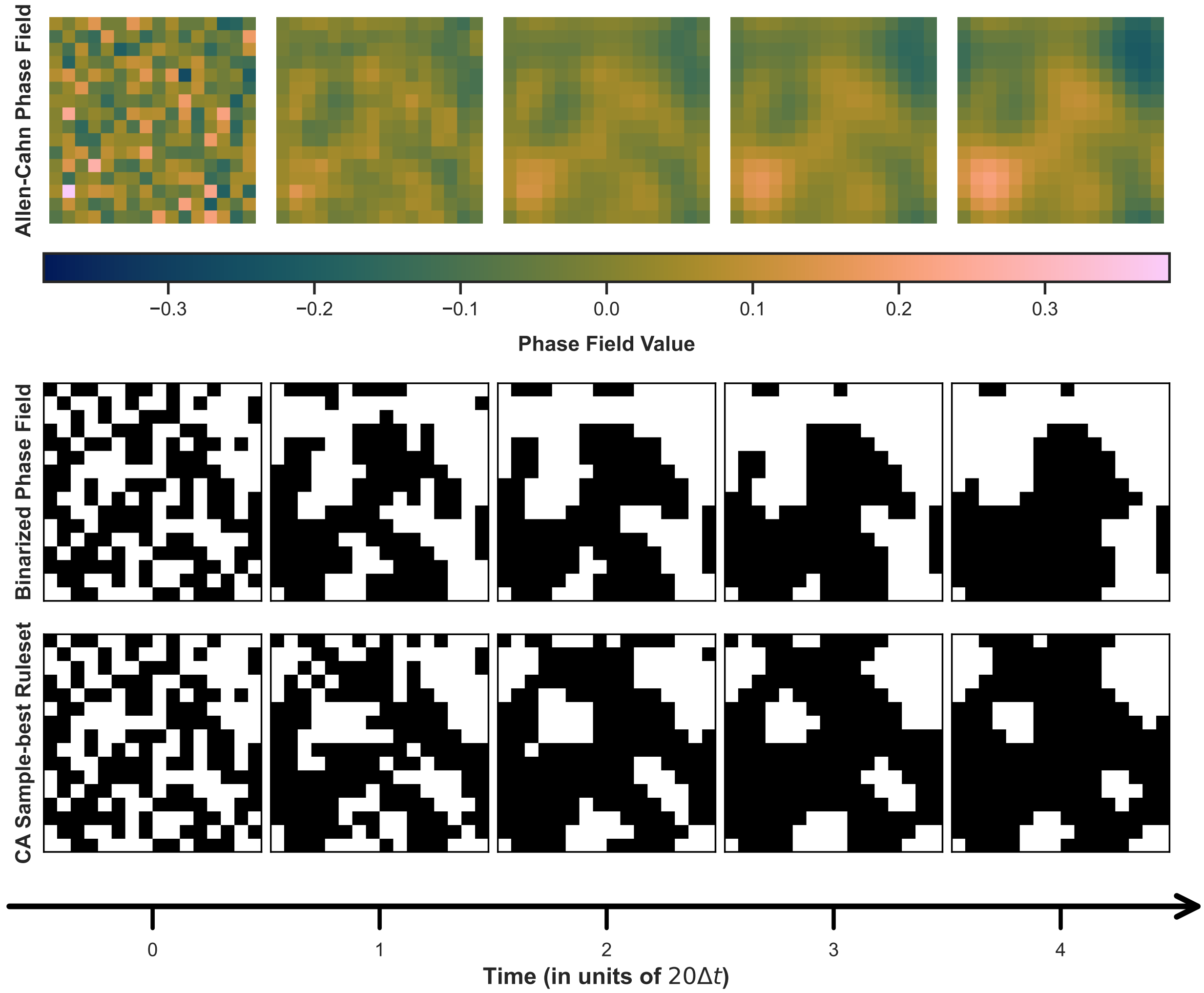

Figure 2: The simulated Allen–Cahn phase field trajectory (starting from a random IC) alongside its binarized version and the trajectory governed by the top-performing inferred CA ruleset. Qualitatively, binarized Allen–Cahn dynamics agree reasonably well with the CA dynamics, as both trajectories shown the same noisy IC evolving into similarly shaped black and white regions.

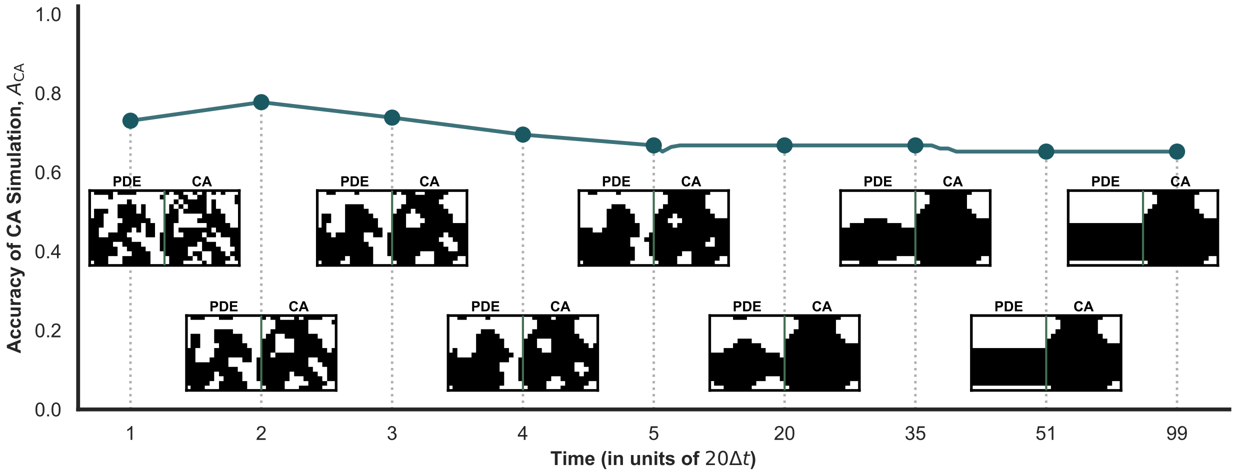

Figure 3: Plot of the cell-wise accuracy (A_{CA}) of the CA simulation relative to the ground-truth binarized Allen–Cahn dynamics; at key timepoints, images of the binarized phase-fields for both simulations are included, labeled as "CA" and "PDE" respectively. A_{CA} is observed to be greater for earlier timepoints, when Allen–Cahn dynamics evolve faster, than for later timepoints, when the Allen–Cahn system is close to equilibrium (complete phase separation) and the CA system stops evolving.

S3.2 Large-scale Simulation

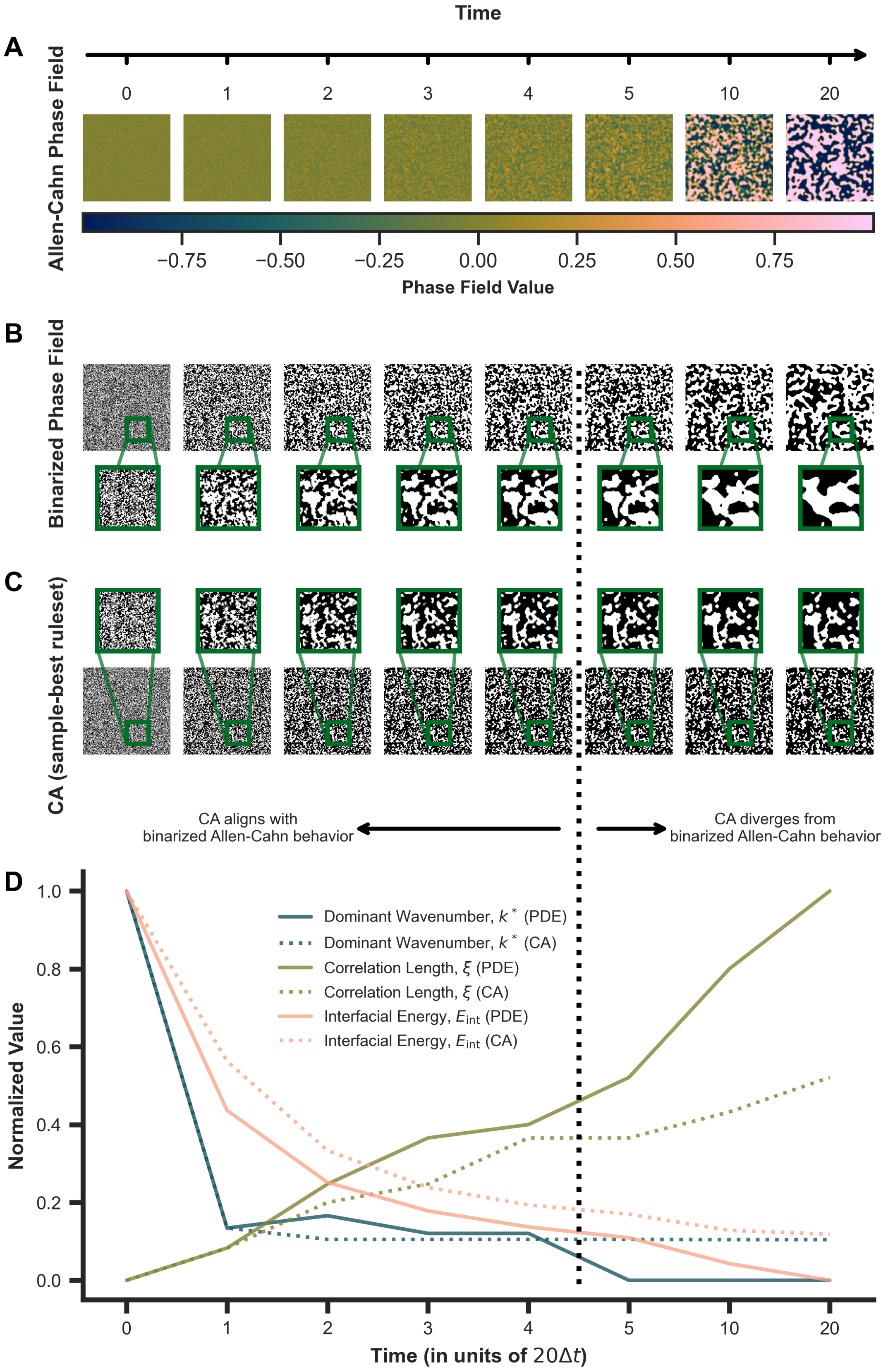

...

Figure 4: Visual comparison of a large-grid (256×256) continuous-valued Allen–Cahn phase field simulation (A), its binarized version (B), and the CA approximation based off a ruleset inferred from a small-grid (16×16) simulation (C). Both continuous-valued and binarized Allen–Cahn simulations are coarse-grained in time using a time spacing parameter $s = 20$. A two-dimensional line-plot comparison of macroscopic observables is included at, where PDE and CA refer to the binarized Allen–Cahn phase field simulation and cellular automaton simulation respectively (D). Visual inspection of large and small-scale behavior and numerical closeness between macroscopic observables indicates agreement between PDE and CA simulations until the time > 4 (where time is in units of $20\Delta t$), after which the macroscopic behavior of the simulations increasingly diverge.

A large-scale simulation from a new, noisy IC was performed to produce a new Allen–Cahn phase separation trajectory (Figure S4A). Comparison between the binarized phase field trajectory (Figure S4B) and the CA simulation (Figure S4C)—computed using the sample-wise best ruleset inferred from the 16×16 trajectory—revealed adequate though imperfect agreement for the first four timesteps and significant disagreement thereafter. The same trend was confirmed quantitatively through an analysis of three macroscopic observables: dominant wavenumber k^*; correlation length ξ; and interfacial energy E_{int} (Figure S4D).

S3.3 Explaining Diverging Dynamics

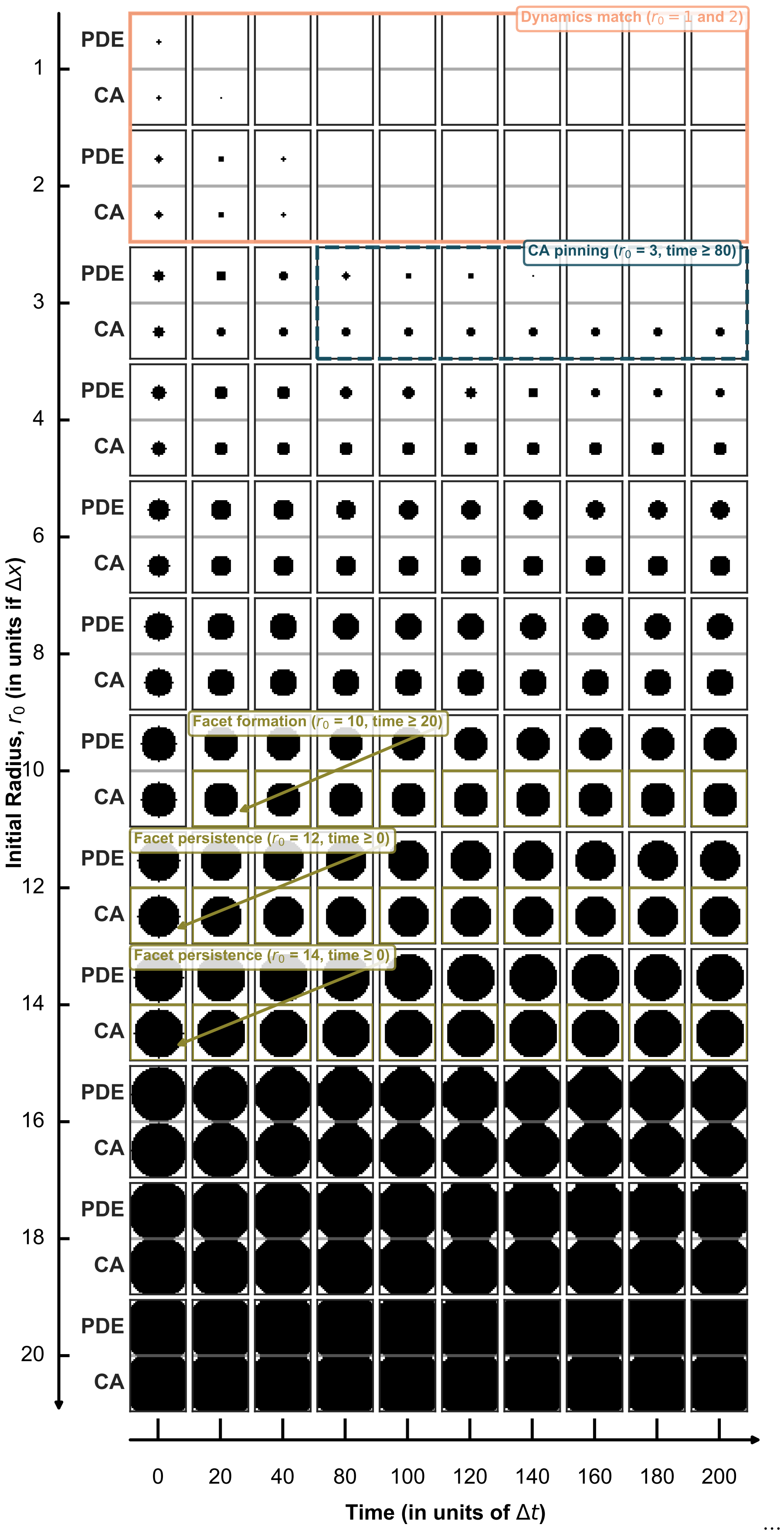

Dynamics match (r_0 = 1 and 2)
CA pinning (r_0 = 3, time ≥ 80)
Facet formation (r_0 = 10, time ≥ 20)
Facet persistence (r_0 = 12, time ≥ 0)
Facet persistence (r_0 = 14, time ≥ 0)
PDE
CA
1
2
3
4
6
8
10
12
14
16
18
20
Initial Radius, r_0 (in units if Δx)
0
20
40
80
100
120
140
160
180
200
Time (in units of Δt)
...

Figure 5: An array of Allen–Cahn dynamics trajectories showing binarized phase field simulations and CA simulations, with rows of snapshots labeled as PDE and CA respectively. Each of the twelve pairs of rows corresponds to an initial "droplet" size, the radius of the circular black region present in each IC (i.e. time = 0 snapshot). Allen–Cahn simulations predict droplet shrinkage and eventual disappearance over time for small initial radii. For very small radii, CA simulations match this behavior qualitatively, though pinning is observed for CA simulations starting from larger droplet radii. Facets also form in CA simulations for larger droplets, indicating anisotropic behavior. For very large droplets, periodic/toroidal boundary conditions enable droplet self-contact and subsequent interface flattening, which occurs in both PDE and CA simulations, though CA simulations slightly deviate.

The main reason our inferred CA rules could not capture the precise dynamics of the Allen–Cahn system, especially for long time durations, was the limitations imposed by non-directional (i.e. isotropic) transition rules. Since, for each cell in the grid, the number of neighboring cells of each possible state is the only information relevant for determining the transition state, there is no way to encode effects based on local curvature. This is because a sharply "curved" set of neighbors (e.g. 3 black cells clustered in the top left of an otherwise white 3×3 kernel) is formally indistinguishable from a "flat" set (e.g. 3 black cells lined up on the bottom of an otherwise white 3×3 kernel). This is evidenced by the "CA pinning" observed in Figure S5, where for droplets of a critically large radius, the CA simulation could not properly simulate droplet shrinkage over time, as the non-directional rules cannot properly distinguish flat versus curved interfaces, only considering numbers of black and white neighbors. Thus, for our CA approximation, curvature could not drive phase separation, and our CA ruleset could only function as a crude approximation of the Allen–Cahn system. Furthermore, the geometry of the CA grid was itself also a limiting factor; the intrinsic neighborhood anisotropy of the square grid's Moore neighborhood resulted in facet formation, at the expense of unphysical sharp interfaces at facet endpoints (Figure S5). This issue also appeared in the numerical Allen–Cahn simulation owing to the discrete Laplacian Equation (S54) but was far more pronounced in the CA simulation due to its binary, rather than continuous, phase field.

S4 Conclusion

We have empirically shown, in a controlled proof-of-concept setting, that AutomataGPT can infer a ruleset that captures salient short-horizon features of discretized and binarized Allen–Cahn phase-separation dynamics. Unfortunately, the intrinsic limitations of 2D, binary, non-directional, deterministic, radius-1 Moore neighborhood CA result in local curvature insensitivity resulting in scale-sensitive pinning effects. Still, the closeness of our CA system to ground-truth on both micro and macro scales for short time durations shows promise and suggests that more complex CA rule spaces might unlock new possibilities for simulating a diverse range of systems.

References

[1] V. L. Ginzburg, L. D. Landau, In V. L. Ginzburg, editor, *On Superconductivity and Superfluidity: A Scientific Autobiography*, 113–137. Springer, Berlin, Heidelberg, ISBN 978-3-540-68008-6, **2009**, URL `https://doi.org/10.1007/978-3-540-68008-6_4`.

[2] S. M. Allen, J. W. Cahn, "A microscopic theory for antiphase boundary motion and its application to antiphase domain coarsening", *Acta Metallurgica* **1979**, *27*, 6 1085.

[3] J. W. Cahn, "On spinodal decomposition", *Acta Metallurgica* **1961**, *9*, 9 795.

[4] J. W. Cahn, J. E. Hilliard, "Free Energy of a Nonuniform System. I. Interfacial Free Energy", *The Journal of Chemical Physics* **1958**, *28*, 2 258.

[5] W. J. Boettinger, J. A. Warren, C. Beckermann, A. Karma, "Phase-Field Simulation of Solidification", *Annual Review of Materials Research* **2002**, *32*, Volume 32, 2002 163.

[6] H. Emmerich, *The Diffuse Interface Approach in Materials Science: Thermodynamic Concepts and Applications of Phase-Field Models*, Springer Science & Business Media, **2004**, google-Books-ID: vZ8MBwAAQBAJ.

[7] M. Abramowitz, I. A. Stegun, *Handbook of mathematical functions: with formulas, graphs and mathematical tables [conference under the auspices of the National science foundation and the Massachussetts institute of technology]*, Dover books on advanced mathematics. Dover publ, New York, unabridged, unaltered and corr. republ. of the 1964 ed edition, **1972**.
