# Supplementary figures and images for: AutomataGPT: Transformer‐Based Forecasting and Ruleset Inference for Two‐Dimensional Cellular Automata
Source: Adv Sci (Weinh). 2026 Apr 9;13(33):e11352. doi: 10.1002/advs.202511352 (PMC13271642; doi:10.1002/advs.202511352)

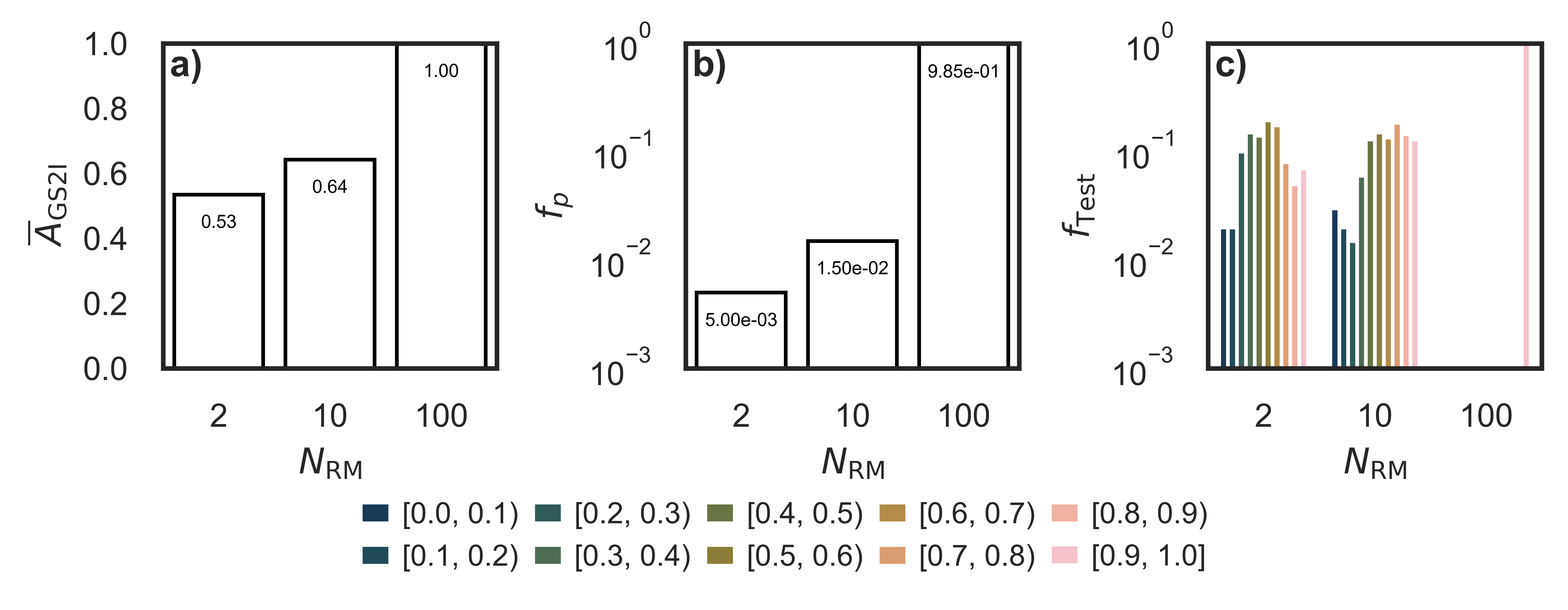

Supplement: Supplementary file 2 — Supporting File 2: advs75040‐sup‐0002‐Data.zip. [file ADVS-13-e11352-s001.zip › AutomataGPT-main/Figures/Automata_GPT_Forward_Models_Accuracy_v_NRM.png]

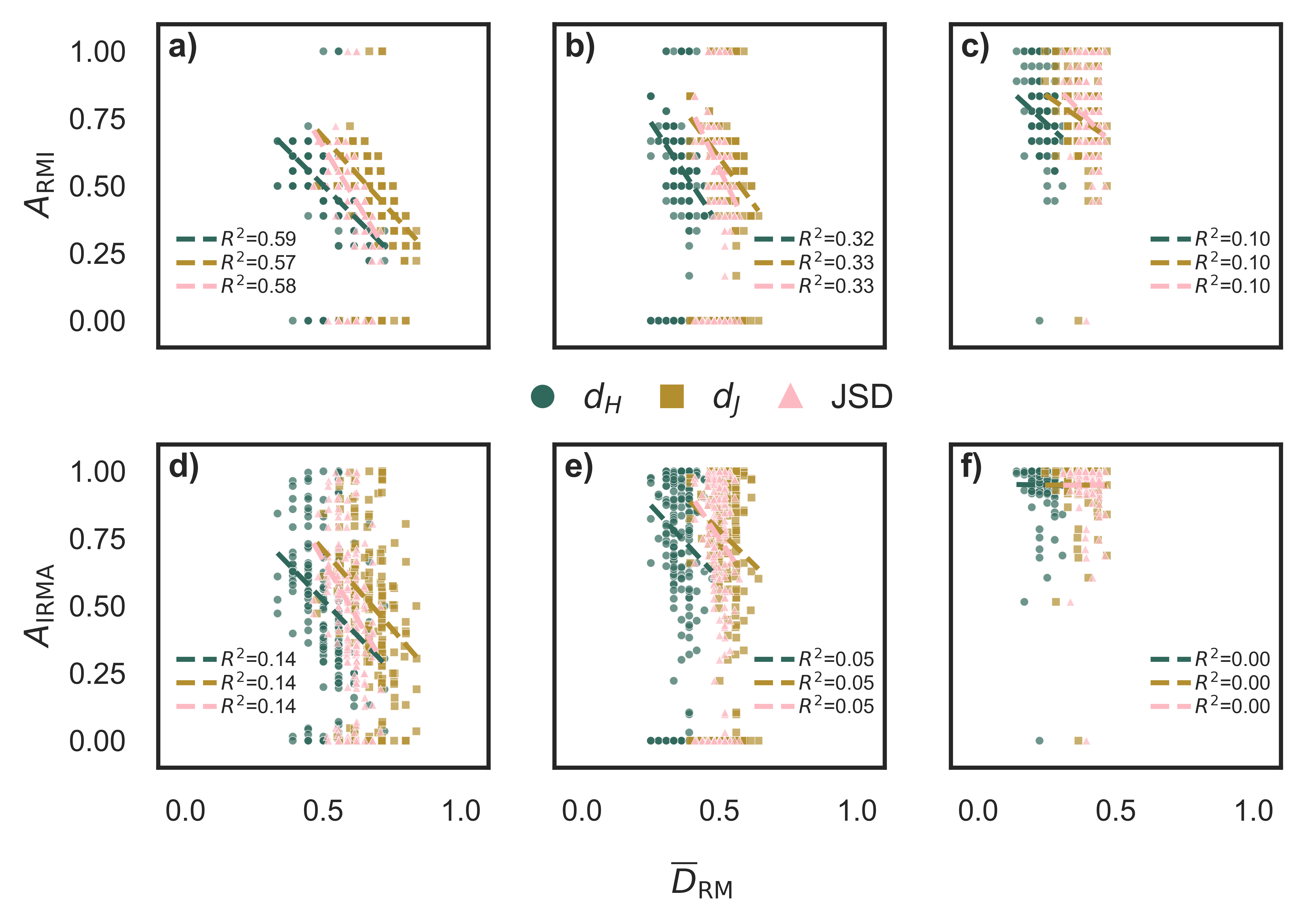

Supplement: Supplementary file 2 — Supporting File 2: advs75040‐sup‐0002‐Data.zip. [file ADVS-13-e11352-s001.zip › AutomataGPT-main/Figures/Automata_GPT_Inverse_Models_Accuracy_v_Matrix_Diff.png]

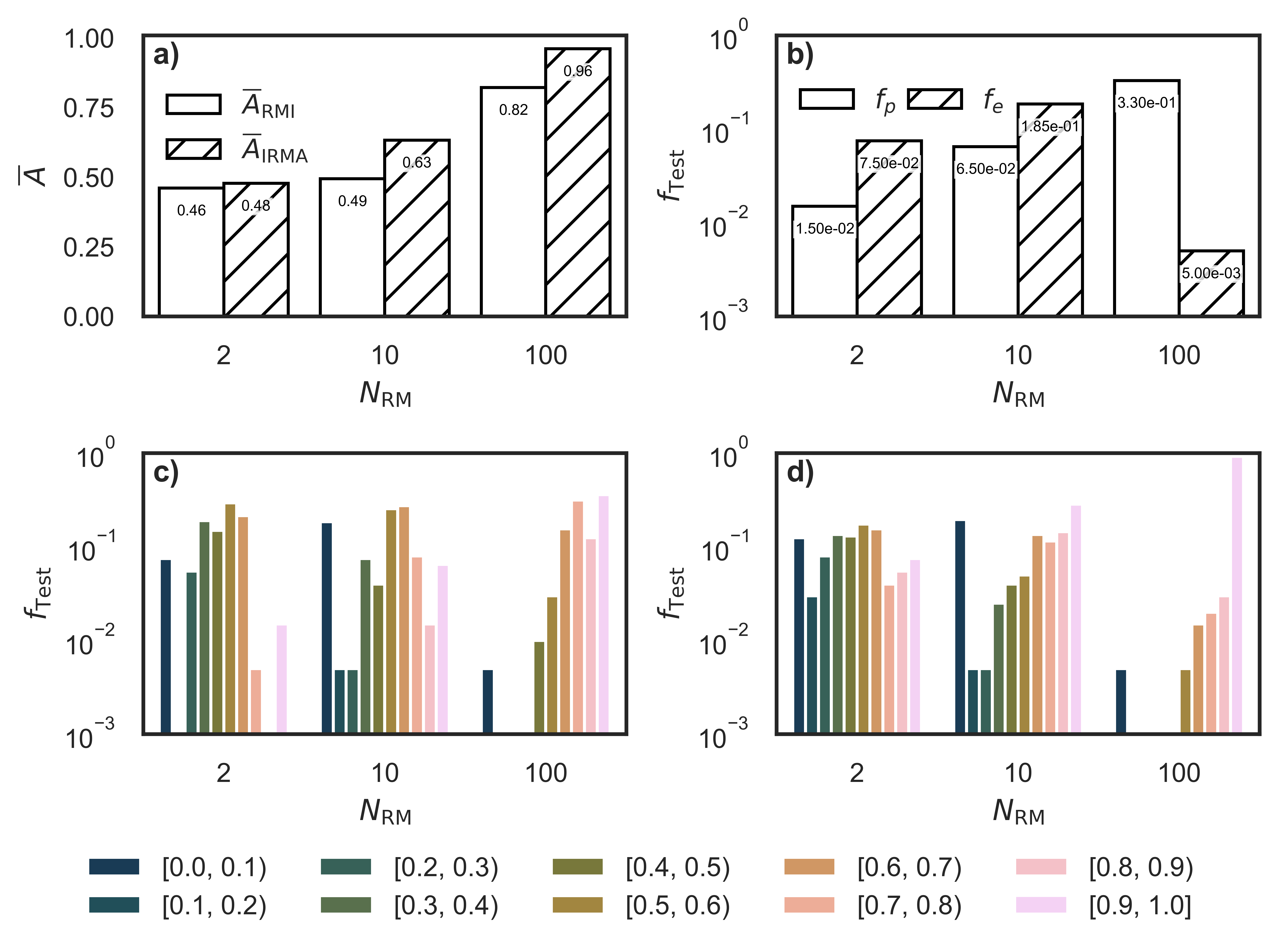

Supplement: Supplementary file 2 — Supporting File 2: advs75040‐sup‐0002‐Data.zip. [file ADVS-13-e11352-s001.zip › AutomataGPT-main/Figures/Automata_GPT_Inverse_Models_Accuracy_v_NRM_COLOR.png]

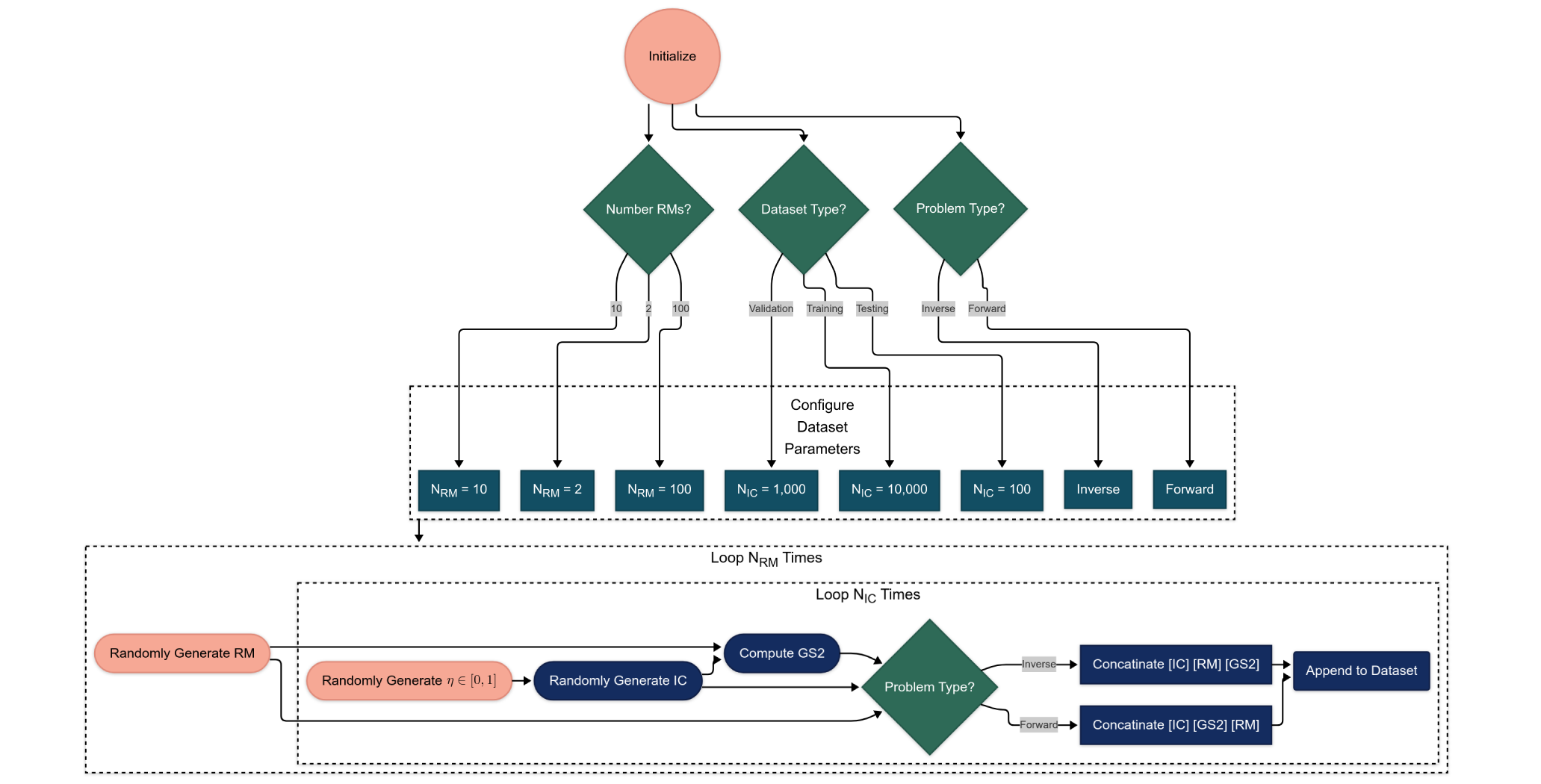

Supplement: Supplementary file 2 — Supporting File 2: advs75040‐sup‐0002‐Data.zip. [file ADVS-13-e11352-s001.zip › AutomataGPT-main/Figures/Dataset_Gen@300ppi.png]

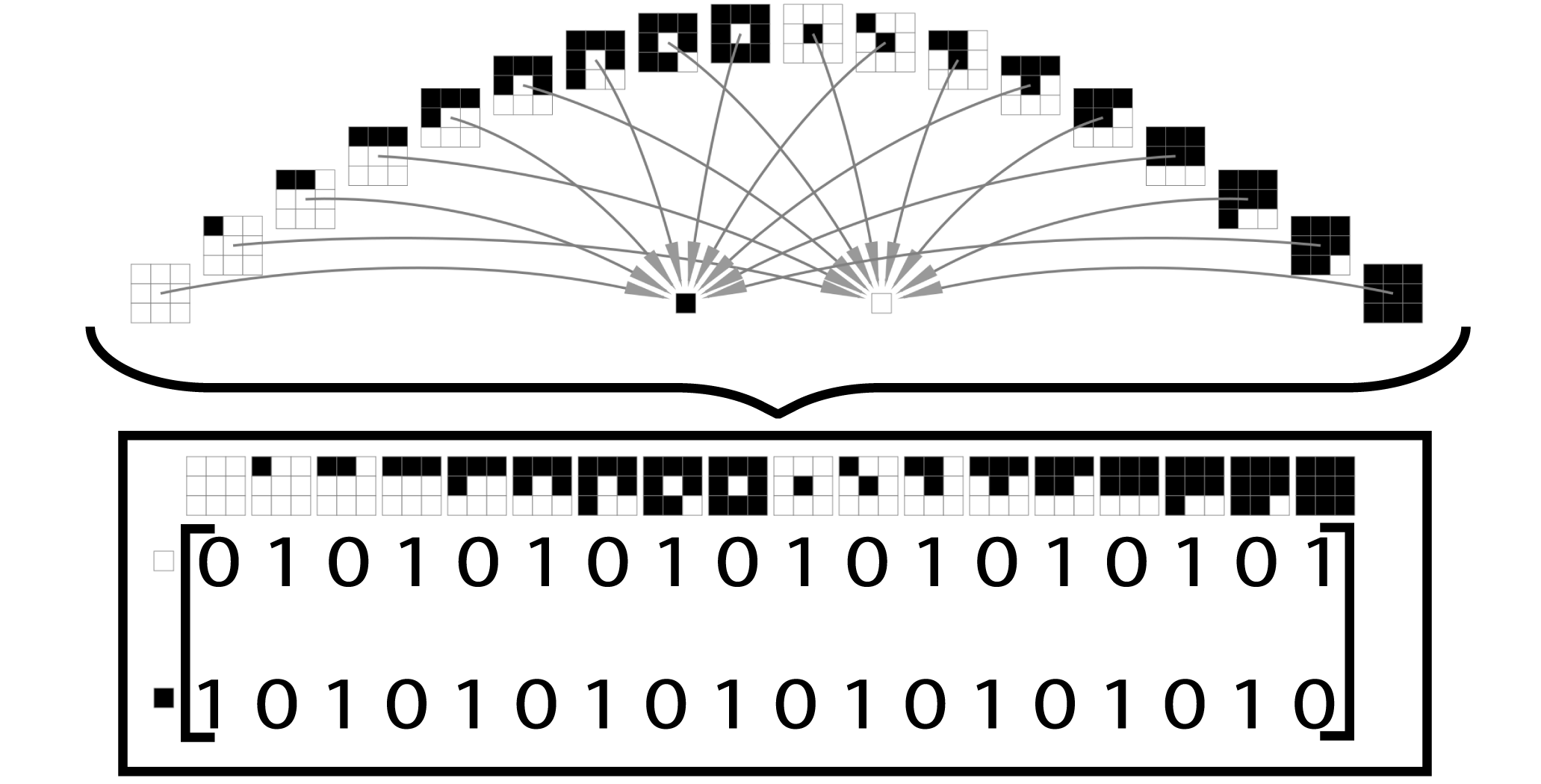

Supplement: Supplementary file 2 — Supporting File 2: advs75040‐sup‐0002‐Data.zip. [file ADVS-13-e11352-s001.zip › AutomataGPT-main/Figures/Graph_to_Matrix.png]

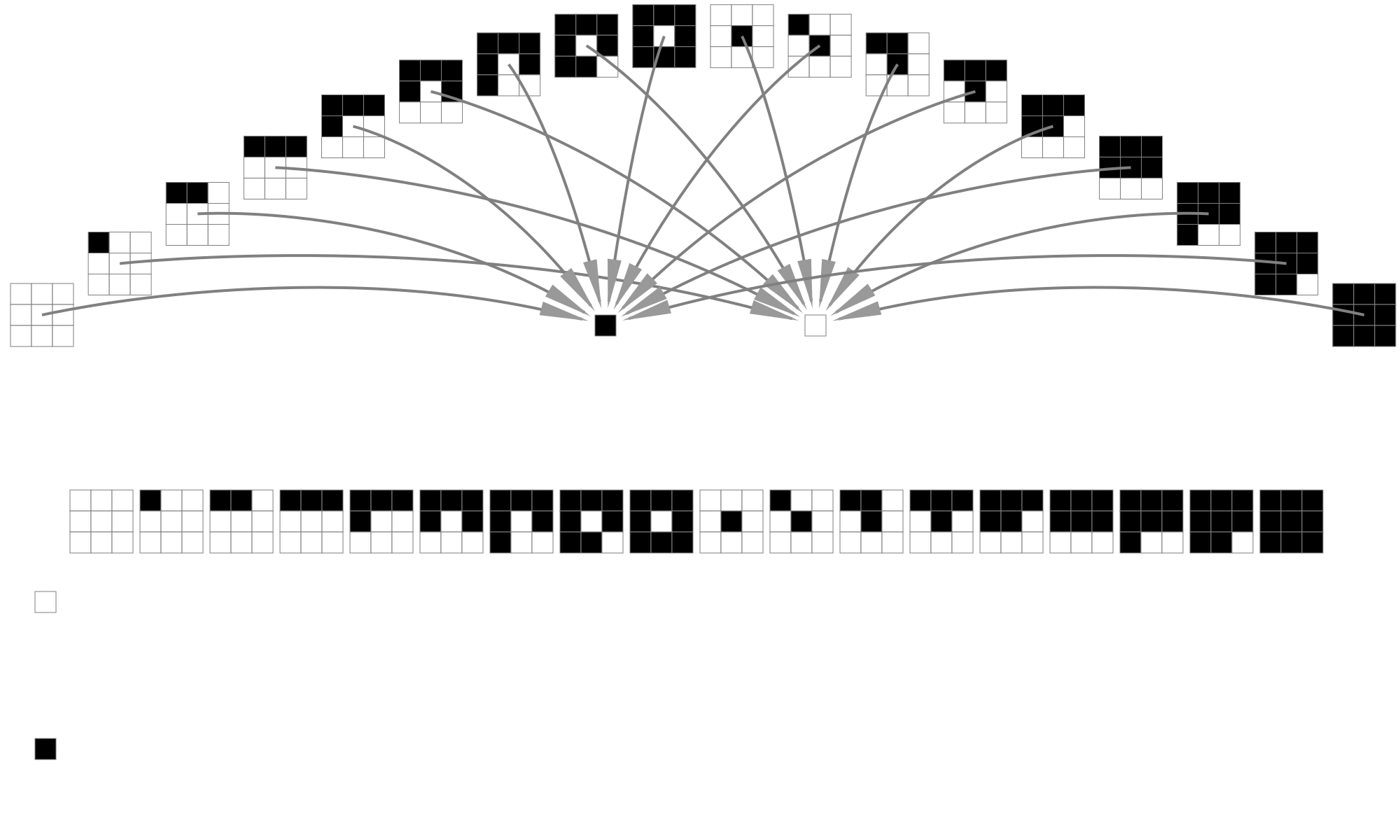

Supplement: Supplementary file 2 — Supporting File 2: advs75040‐sup‐0002‐Data.zip. [file ADVS-13-e11352-s001.zip › AutomataGPT-main/Figures/semicircle_cellular_automata_rules.png]
